# Multifunctional Glycoconjugates for Recruiting Natural Antibodies against Cancer Cells

**DOI:** 10.1002/chem.201903327

**Published:** 2019-10-15

**Authors:** Benjamin Liet, Eugénie Laigre, David Goyard, Biagio Todaro, Claire Tiertant, Didier Boturyn, Nathalie Berthet, Olivier Renaudet

**Affiliations:** ^1^ DCM, UMR 5250 Université Grenoble Alpes, CNRS 38000 Grenoble France

**Keywords:** antibodies, cancer, click chemistry, glycoconjugates, multivalency

## Abstract

We have developed a fully synthetic and multifunctional antibody‐recruiting molecule (ARM) to guide natural antibodies already present in the blood stream against cancer cells without pre‐immunization. Our ARM is composed of antibody and tumor binding modules (i.e., ABM and TBM) displaying clustered rhamnose and cyclo‐RGD, respectively. By using a stepwise approach, we have first demonstrated the importance of multivalency for efficient recognition with naturel IgM and α_v_β_3_ integrin expressing M21 tumor cell line. Once covalently conjugated by click chemistry, we confirmed by flow cytometry and confocal microscopy that the recognition properties of both the ABM and TBM are conserved, and more importantly, that the resulting ARM promotes the formation of a ternary complex between natural IgM and cancer cells, which is required for the stimulation of the cytotoxic immune response in vivo. Due to the efficiency of the synthetic process, a larger diversity of heterovalent ligands could be easily explored by using the same multivalent approach and could open new perspectives in this field.

## Graphical abstract

**Synthetic commander**: A potent synthetic and multifunctional antibody‐recruiting molecule is synthesized through the combination of antibody and tumor binding modules, thereby providing a candidate that promotes the formation of a ternary complex between natural antibodies and cancer cells, which is required for the stimulation of the cytotoxic immune response in vivo.

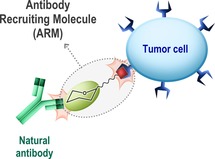

## Introduction

Despite significant progress in surgery, chemo‐ and radiotherapy, cancer treatments still require the development of novel strategies improving long‐term survival and tolerability. If immunotherapy approaches, such as checkpoint inhibitors, adoptive cell transfer, vaccines, monoclonal antibodies, or cytokines are revolutionizing this field, these strategies still remain inefficient in many cases and suffer from critical drawbacks including side effects, pharmacokinetics, production and selectivity issues.^1^, ^2^ The utility of synthetic chemistry has been demonstrated in this field over the past decades through the development of therapeutic vaccines.^3^, ^4^ More recently, several groups have demonstrated that fully synthetic bifunctional molecules, named antibody recruiting molecules (ARMs), have the ability to redirect natural antibodies (Abs) against cancer cells to promote immune‐mediated cytotoxicity.^5^, ^6^ Also used successfully against pathogens,^7^, ^8^, ^9^, ^10^ this concept is based on the covalent association of two distinct recognition domains, one for the antibodies present in humans and one for a cancer‐specific biomarker, thus enabling simultaneous binding of both Abs and cancer cells. For example, Kiessling and co‐workers^11^ described an ARM containing a RGD peptidomimetic as ligand for α_v_β_3_ integrins and the α‐Gal trisaccharide as specific epitope for a large class of Abs present in the bloodstream of humans. This molecule was able to inhibit integrin‐mediated cell adhesion by creating multivalent contacts at the cell surface and to recruit natural Abs from human serum to mediate cytotoxicity against tumors. In another study, a bifunctional molecule containing nitrophenol to bind endogenous IgM and the modified NeuAcα2‐6Galβ1‐4GlcNAc trisaccharide to target CD22‐expressing B‐cell lymphomas has been described.^12^ It was demonstrated that this ligand uses IgM as a protein scaffold to bind on the surface of B‐cells in a multivalent fashion to promote immune response. More recently, the group of Spiegel designed a small ARM directed against prostate tumors.^13^ This compound, which contains a glutamate urea moiety for targeting the prostate specific membrane antigen and dinitrophenol as Abs epitope was able to stimulate cytotoxicity in the presence of anti‐DNP antibodies and peripheral blood mononuclear cells. Besides being highly promising, these pioneering studies and a more recent study with polymeric structure^14^ clearly pointed out the requirement of the multivalent presentation of ligands to stimulate a potent immunological response. Here, we report the first generation of functional ARM molecules displaying well‐defined clusters of both antibody and tumor binding modules. For this purpose, the access of a high‐molecular weight ARM was achieved through stepwise biomolecular assemblies by using click chemistry (CuAAC). We demonstrated by flow cytometry and confocal microscopy experiments the formation of ternary complexes between the targeted cancer cell and natural antibodies, which is a prerequisite for the stimulation of cytotoxic response.^5^, ^6^

## Results and Discussion

### Antibody binding module (ABM)

**Scheme 1:**
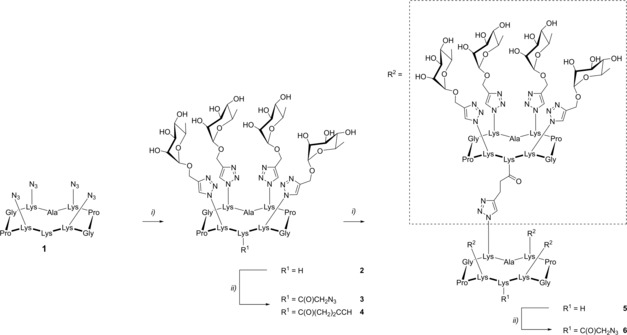
Synthesis of tetra‐ and hexadecavalent rhamnosylated ABMs. Reagents and conditions: i) Propargyl α‐l‐rhamnopyranoside,^21^ CuSO_4_ **⋅**5 H_2_O, tris(3‐hydroxypropyltriazolylmethyl)amine (THPTA), NaAsc, DMF/phosphate‐buffered saline (PBS), RT, 1 h; ii) azidoacetic^22^ or pentynoic acid succinimide ester,^23^ diisopropylethylamine (DIPEA), DMF, RT, 1 h (see the Supporting Information).

To design ARMs, the first crucial parameter to consider is the recognition of natural circulating Abs, such as IgG and IgM, which are present in different ratio in sera of all individuals during their life. One important features of these Abs is their ability to interact with multiple antigens, in particular carbohydrates. High‐throughput glycan arrays of human sera have allowed the identification of antigens in healthy donors, such as l‐rhamnose (Rha), which are known to bind to Abs of a broad class of population.^15^, ^16^ However, this screening did not take into consideration the fact that immunoglobulins are present in diverse multimeric isoforms and that the “glycoside‐cluster effect”^17^ may strongly impact both affinity and selectivity of the Ab/antigen binding. Moreover, multivalent presentation of glycans may also be better suited for patients having lower values of circulating antibodies. Therefore, we decided to synthesized tetra‐ and hexadecavalent Rha‐based ABMs (Scheme 1) by using peptide carriers previously identified as well‐suited scaffolds for multivalent presentation of sugars.^18^, ^19^ Starting from the tetraazido cyclodecapeptide **1**,^20^ a first CuAAC reaction with propargyl l‐rhamnopyranoside^21^ afforded the tetravalent cluster **2**. On one hand, the introduction of azidoacetic acid on the remaining free lysine residue led to the tetravalent ABM **3**. On the other hand, functionalization of this lysine with pentynoic acid allowed for a second CuAAC reaction. Intermediate **4** was reacted with **1**, yielding dendrimer **5**. A final lysine functionalization with azidoacetic acid led to the hexadecavalent ABM **6**.

**Figure 1:**
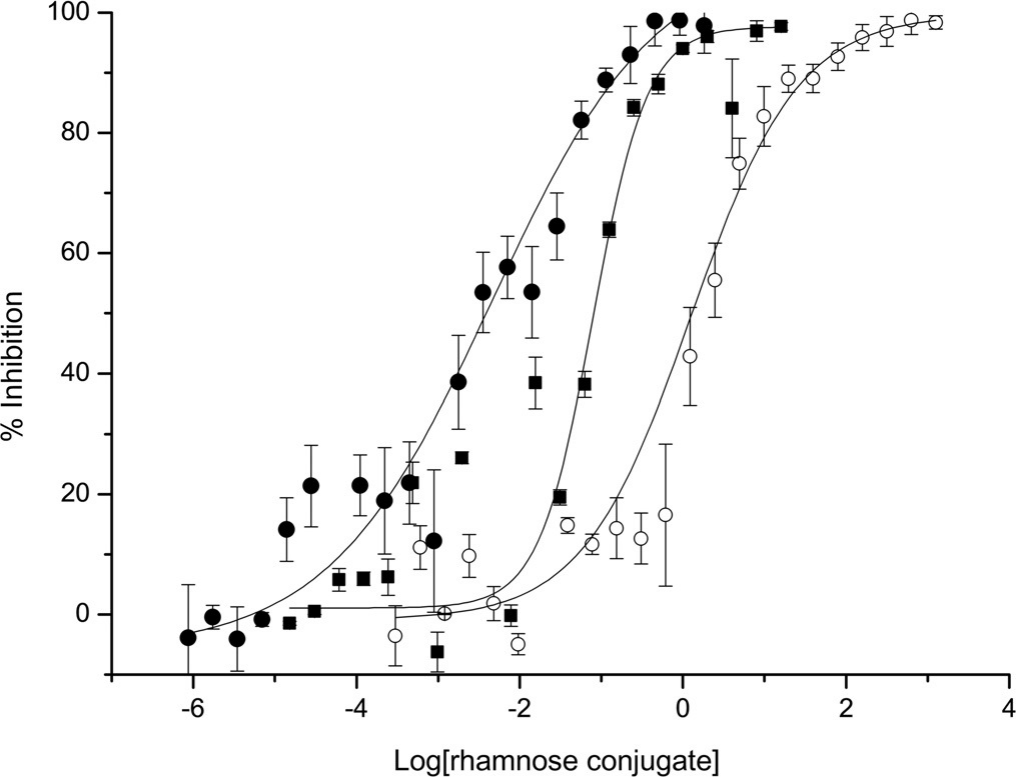
Competitive binding assay with human serum and monovalent Rha (○), tetravalent (▪, **3**) and hexadecavalent (•, **6**) conjugates. The assay measures the inhibition of the binding of human anti‐Rha IgM to Rha coated on microplate wells (PAA‐Rha). The binding was revealed with Alexa‐Fluor488‐labeled goat anti‐human IgM. Inhibition percentage versus the logarithm of the Rha conjugate concentration (in mm) is plotted. Data reported are an average of three independent experiments.

We next performed enzyme‐linked immunosorbent assay (ELISA)‐type studies to evaluate the ability of glycoconjugates **3** and **6** to bind natural Abs. Microtiter plates were first coated with a commercial Rha‐functionalized poly[*N*‐(2‐hydroxyethyl)acrylamide] polymer (PAA‐Rha) and incubated with human serum, a source of anti‐Rha antibodies, then fluorescent goat anti‐human IgM and IgG. Interestingly, this experiment first allowed to confirm the presence of anti‐Rha IgM whereas IgG were not detectable in the serum in the tested conditions (Figure S39 in the Supporting Information). This observation is in good agreement with previous studies showing strong ratio variations of circulating antibodies in human sera.^15^, ^16^ A competitive assay was next performed with glycoconjugates **3** and **6** as inhibitors and Rha as the monovalent control (Figure 1).

We determined an IC_50_ value of 84 μm for the tetravalent glycocluster **3** and of 4 μm for the hexadecavalent glycodendrimer **6**, which corresponds, respectively to a 14‐ and 300‐fold inhibitory improvement compared to Rha (IC_50_=1.2 mm). As expected, this result thus confirmed that multimeric presentation of Rha ensures more efficient binding with IgM certainly due to the glycoside cluster effect commonly observed with lectins.

### Tumoral binding module (TBM)

**Scheme 2:**
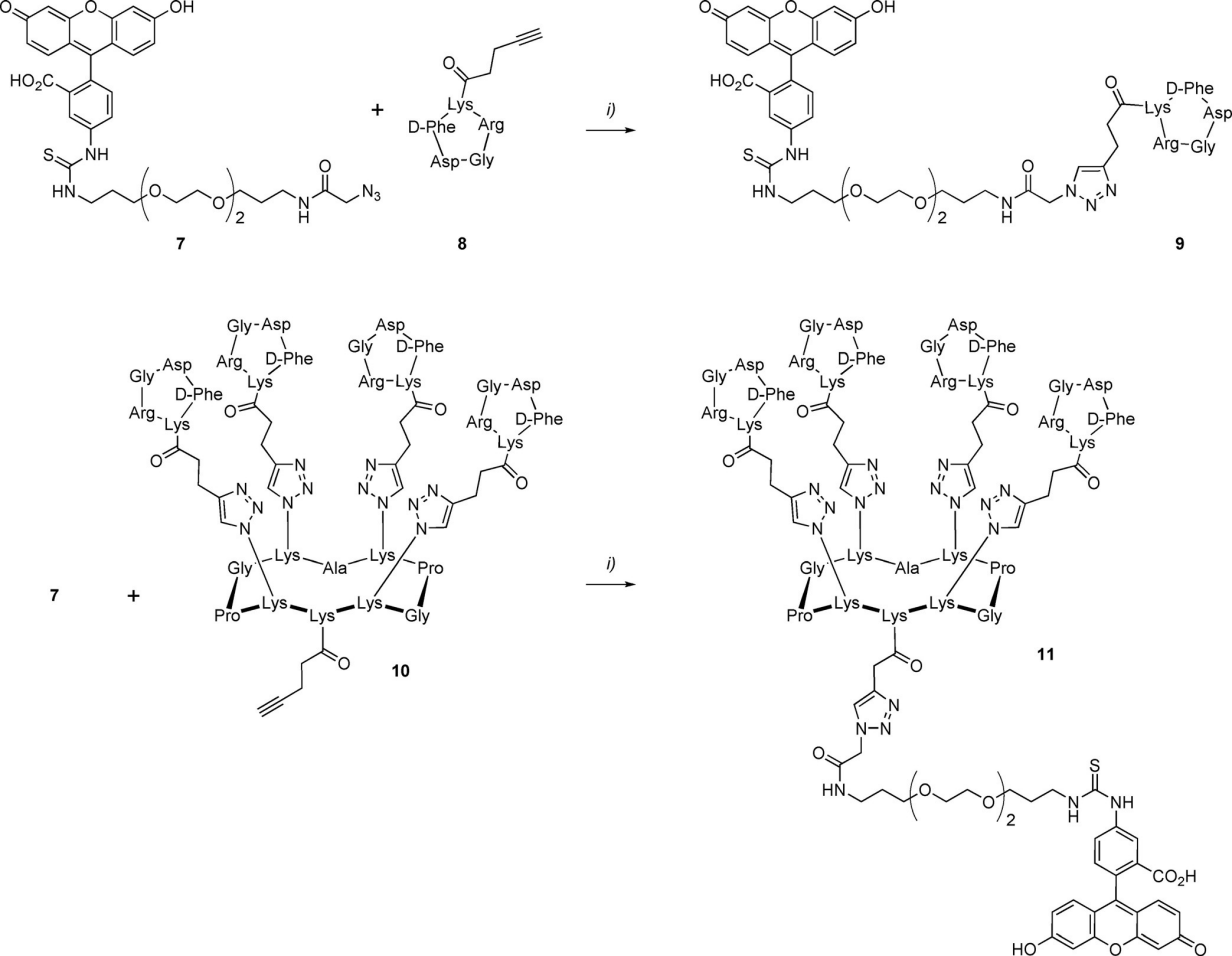
Synthesis of mono‐ and tetravalent fluorescent RGD‐based conjugates. Reagents and conditions: i) CuSO_4_⋅5 H_2_O, THPTA, NaAsc, DMF/PBS, RT, 1 h (see the Supporting Information).

Targeted therapy aims at delivering drugs in tumor cells by using selective ligands of receptors present at their surface.^24^ We selected a well‐known cyclopeptide containing the triad sequence Arg‐Gly‐Asp (cRGD), which is one of the most potent ligand for the α_v_β_3_ integrins that are overexpressed in tumor microenvironment.^25^ We previously demonstrated that the multivalent presentation of cRGD onto a cyclopeptide scaffold provides an efficient tumor delivery system and imaging agent especially for fluorescence‐guided surgery.^26^, ^27^ Here, we synthesized fluorescent mono‐ and tetravalent RGD‐based conjugates (Scheme 2) to evaluate their binding potency. The monovalent compound **9** was prepared through a CuAAC reaction between the fluorescent compound **7** and the cRGD derivative **8**.^28^ The tetravalent conjugate **11** was also obtained by a CuAAC coupling between **7** and the alkyne‐functionalized scaffold **10**.

**Figure 2:**
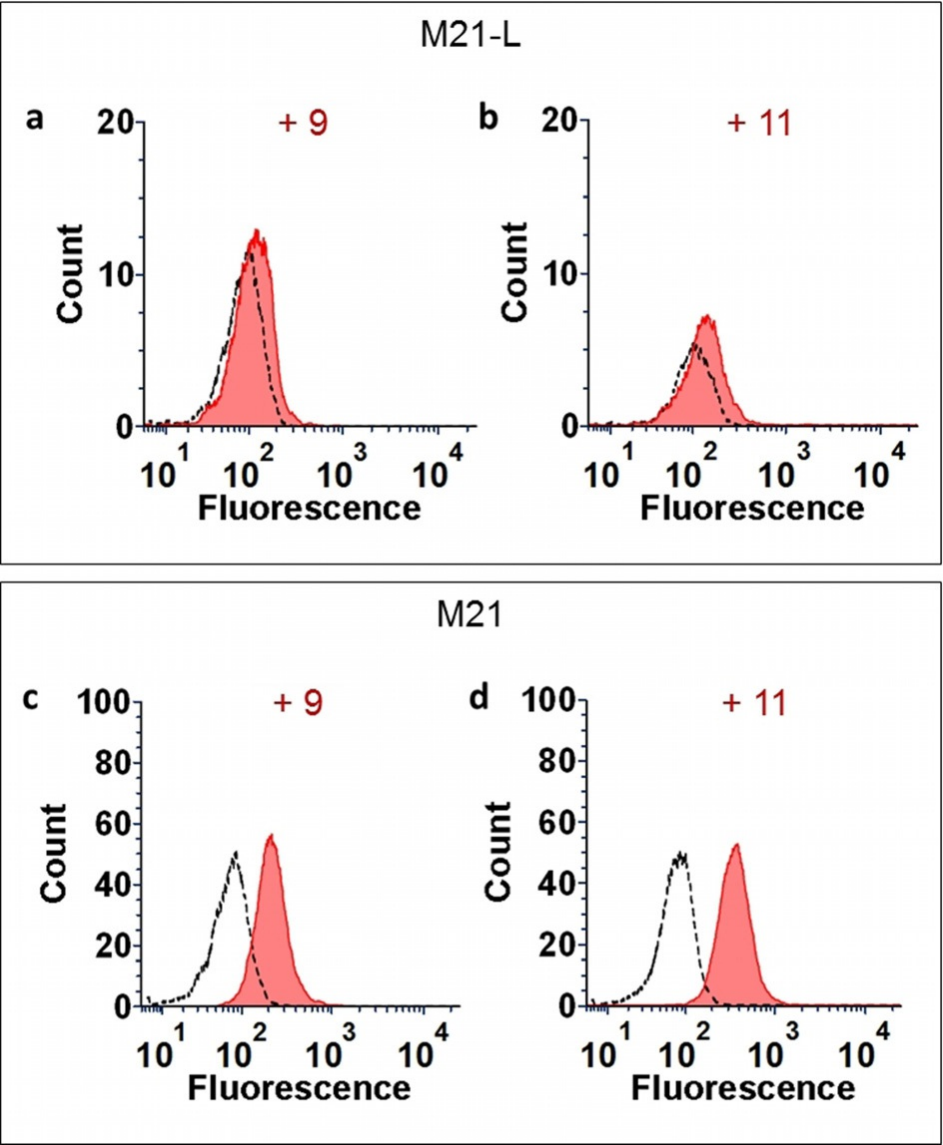
Evaluation of the binding of the fluorescent TBMs **9** and **11** (5 μm, red) to M21 (high levels of integrins) and M21‐L (low levels of integrins) cells by flow cytometry. a) Binding of **9** to M21‐L. b) Binding of **11** to M21‐L. c) Binding of **9** to M21. d) Binding of **11** to M21. The autofluorescence of the cells is represented with dashed lines.

We next assessed the recognition potency of the fluorescein isothiocyanate (FITC)‐labelled TBMs **9** and **11** by flow cytometry with two melanoma cells, M21 which expresses α_v_β_3_ integrins and M21‐L as the negative cell line control (Figure 2). After having confirm the low expression of α_v_β_3_ on M21‐L (Figure S40 in the Supporting Information), we incubated a solution (5 μm) of the monovalent and tetravalent compounds **9** and **11** with both cell lines. As observed previously with similar compounds,^28^ we confirmed the higher potency of the clustered cRGD than the monovalent peptide to bind to M21 cells (Figures 2 c and d). In addition, only low unspecific binding was observed with M21‐L (Figures 2 a and b). Therefore, scaffold **11** was selected as the TBM to be combined with Rha‐based ABMs.

### Antibody‐recruiting molecule (ARM)

**Scheme 3:**
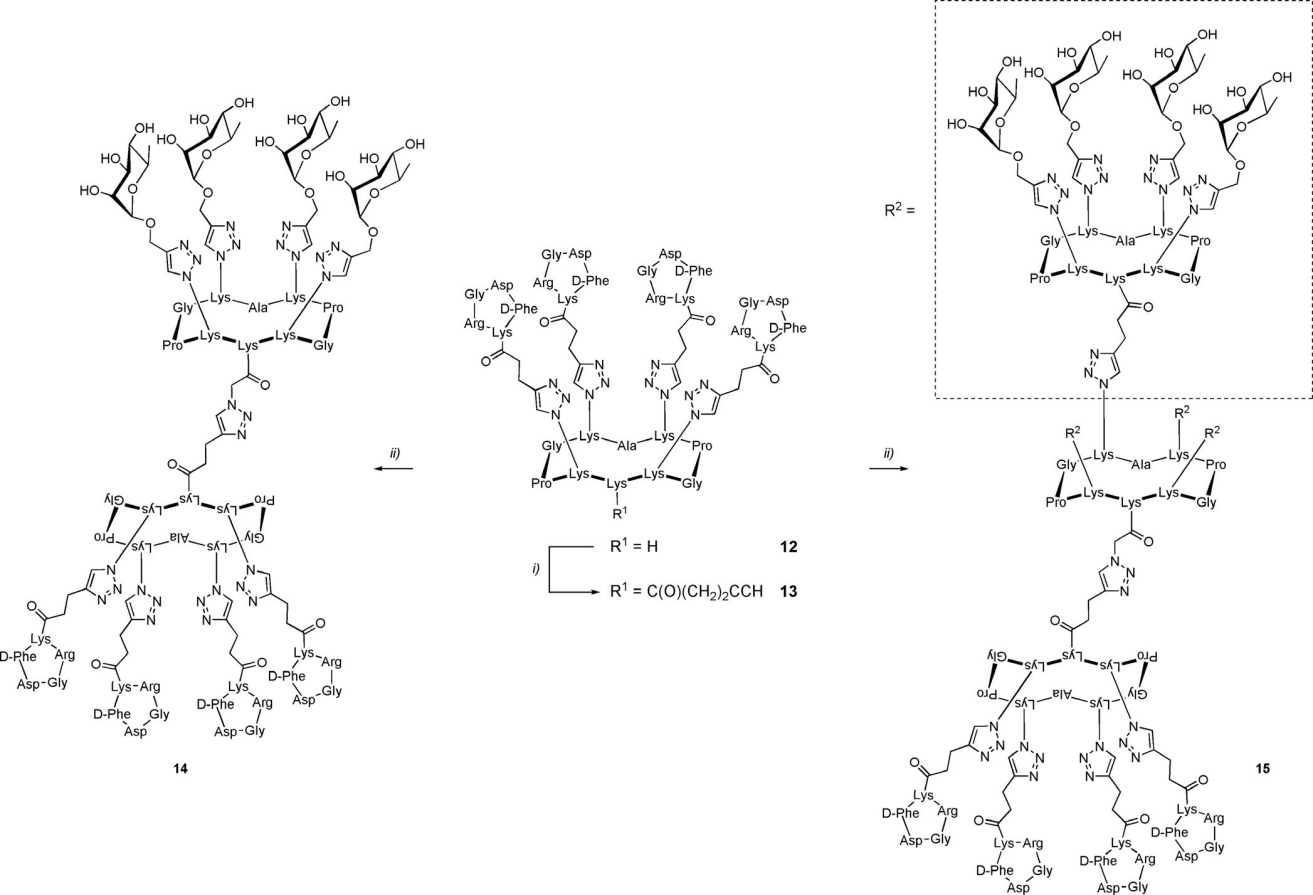
Synthesis of antibody‐recruiting molecules. Reagents and conditions: i) Pentynoic acid succinimide ester, DIPEA, DMF, RT, 1 h; ii) CuSO_4_ **⋅**5 H_2_O, THPTA, NaAsc, DMF/PBS, RT, 1 h (see the Supporting Information).

After having confirmed the recognition potency of ABMs and TBMs, we finally combined them covalently into a single molecule to provide fully synthetic ARMs with recognition properties towards both antibodies and tumors. We constructed several combinations of ARMs displaying either four or sixteen copies of Rha with four copies of cRGD by using a convergent approach as shown in Scheme 3. First, the lysine side chain of the tetravalent cRGD‐based cluster **12** was functionalized with pentynoic acid affording building block **13**. Then, CuAAC reaction with the ABM **3** led to the ARM **14** displaying four copies of both l‐rhamnose and cRGD. The same reaction with ABM **6** afforded compound **15** displaying for copies of cRGD and sixteen l‐rhamnose moieties. These final compounds were characterized by ^1^H NMR spectroscopy, HPLC, and mass spectrometry ensuring their monodispersity and molecular definition.

**Figure 3:**
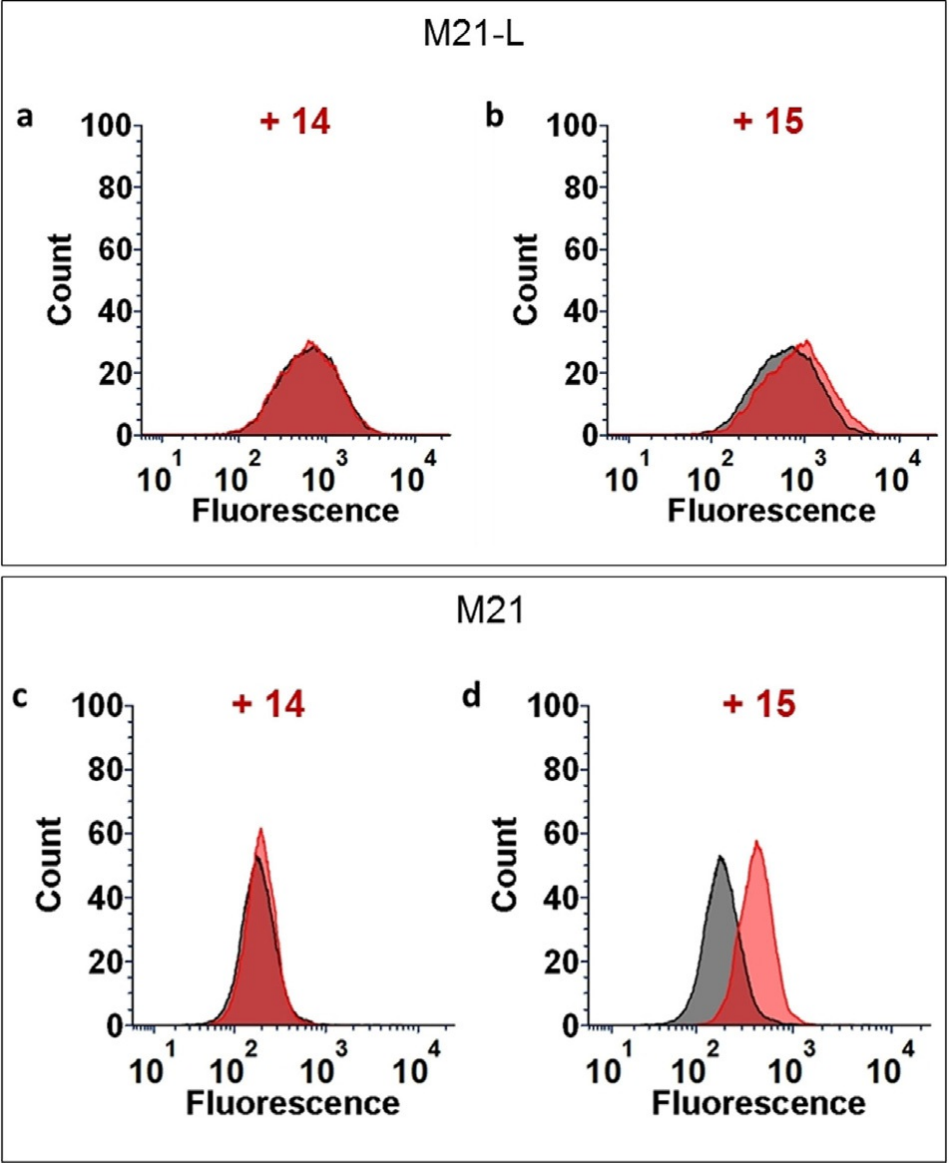
Evaluation by flow cytometry of the binding of ARMs **14** and **15** (100 nm) to M21 and M21‐L cell lines by using a purified rabbit anti‐Rha IgG antibody (10 μg mL^−1^) and revealed with a PE‐coupled anti‐rabbit secondary antibody (1:100). a) Binding of **14** to M21‐L. b) Binding of **15** to M21‐L. c) Binding of **14** to M21. d) Binding of **15** to M21. Controls without the ARM molecules are represented in black.

Past studies demonstrated that ARMs must bind simultaneously both the tumor cell surface and the Abs to form the ternary complex, which is required for immune activation.^5^, ^6^ Therefore, to confirm that the association of ABM and TBMs did not alter their recognition properties, we used an in vitro fluorescent‐based assay to analyze the binding of our final ARM molecules to the selected cells. Flow cytometry and confocal microscopy by using purified anti‐Rha rabbit IgG antibodies and phycoerythrin (PE)‐coupled secondary anti‐rabbit IgG antibody have thus been performed (Figure 3). To this end, M21 and M21‐L cell lines have been successively incubated with various low concentrations of ARMs and these antibodies to prevent the autoinhibition effect that can occur in three‐component binding systems.^29^ Although no binding was detected with M21‐L control cells whatever the ARM used, we indeed observed a fluorescence increase on the M21 cell line overexpressing α_v_β_3_ integrins, implying the formation of a ternary complex with ARM **15** (100 nm) (Figure 3 d). In addition, as determined by ELISA, no binding occurred when a tetravalent ABM is used, which again indicates the importance of hexadecavalent presentation of Rha for the binding with anti‐Rha antibodies.

**Figure 4:**
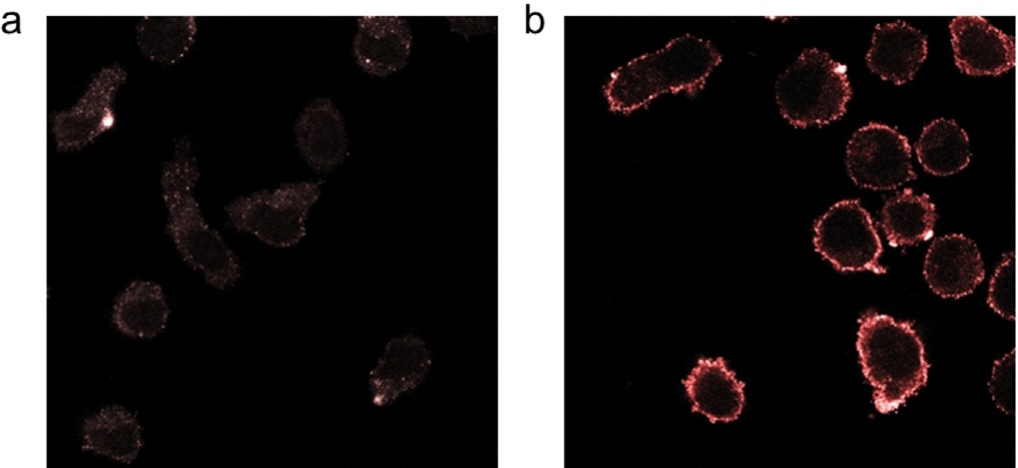
Confocal fluorescence microscopy experiment after incubation with or without the ARM **15** and purified rabbit anti‐Rha antibody (10 μg mL^−1^). The binding was revealed with a PE‐coupled anti‐rabbit secondary antibody (1:100). a) Control experiment without ARM. b) Experiment performed in the presence of the ARM **15** (100 nm).

This result was also confirmed by visualizing the cells from the same experiment under a confocal fluorescence microscope, which clearly allows to observe the localization of the ARM **15** at the cell surface of M21 but not of the M21‐L control cell line (Figure 4). This result clearly demonstrates the accessibility of the ARM **15** to endogenous human antibodies.

**Figure 5:**
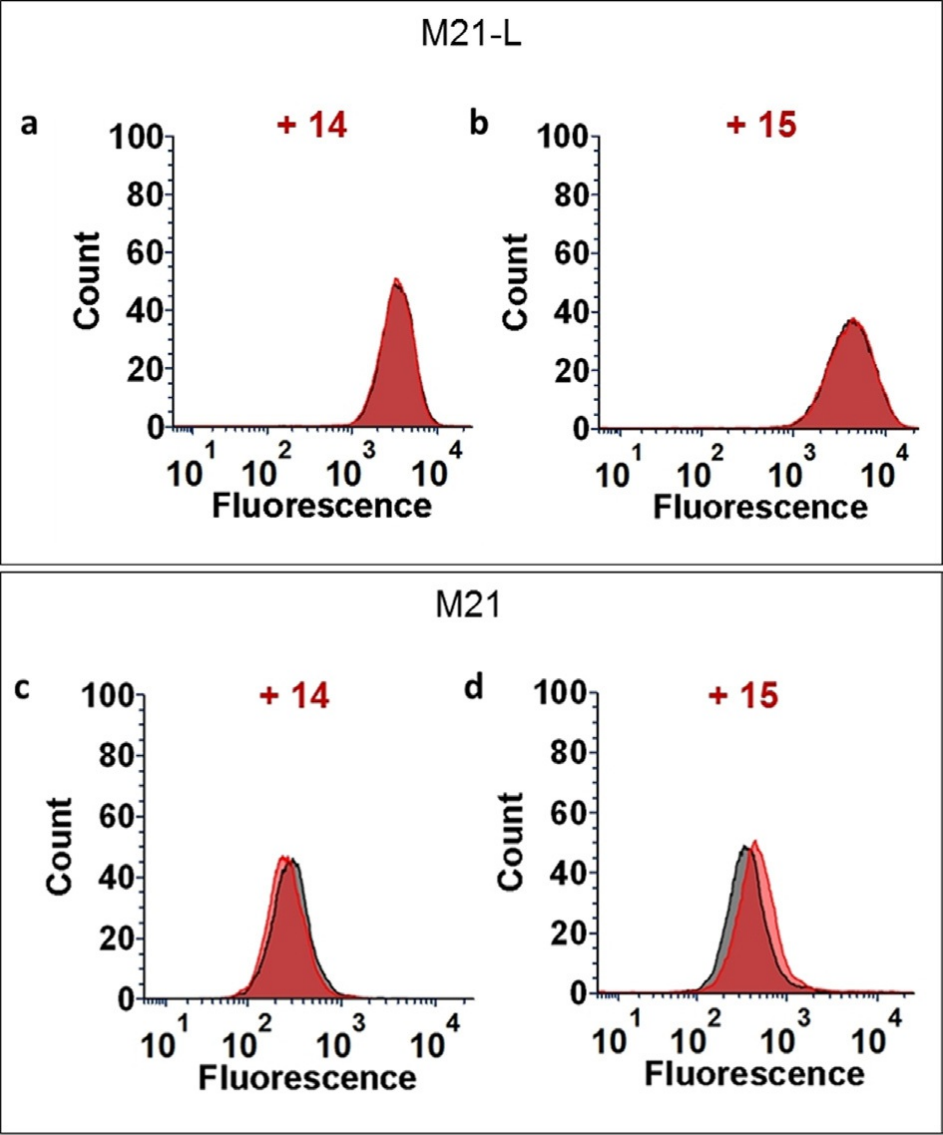
Evaluation of the recruitment of anti‐Rha antibodies present in human serum by flow cytometry with M21 and M21‐L cell lines revealed by binding with Alexa‐Fluor488‐coupled anti‐human IgM antibody (1:400). a) Binding of **14** (100 nm) to M21‐L; b) Binding of **15** (100 nm) to M21‐L; c) Binding of **14** (100 nm) to M21; d) Binding of **15** (100 nm) to M21. Controls without the ARMs molecules are represented in black.

Ideally, ARMs should be delivered without pre‐immunization due to the weakened immune system of cancer patients which could strongly limits the ARM efficiency. For this reason, one crucial step is the demonstration that ARM molecules could be used with human serum as unique source of antibodies. Therefore, similar binding experiments have been performed with the human serum used for ELISA assays in which we observed the presence of anti‐Rha IgM. Flow cytometry experiments first indicated that the ARM **15** only, that is, with the higher number of Rha units, is able to redirect IgM against M21 cells overexpressing integrins whereas M21‐L remained unbound (Figure 5).

**Figure 6:**
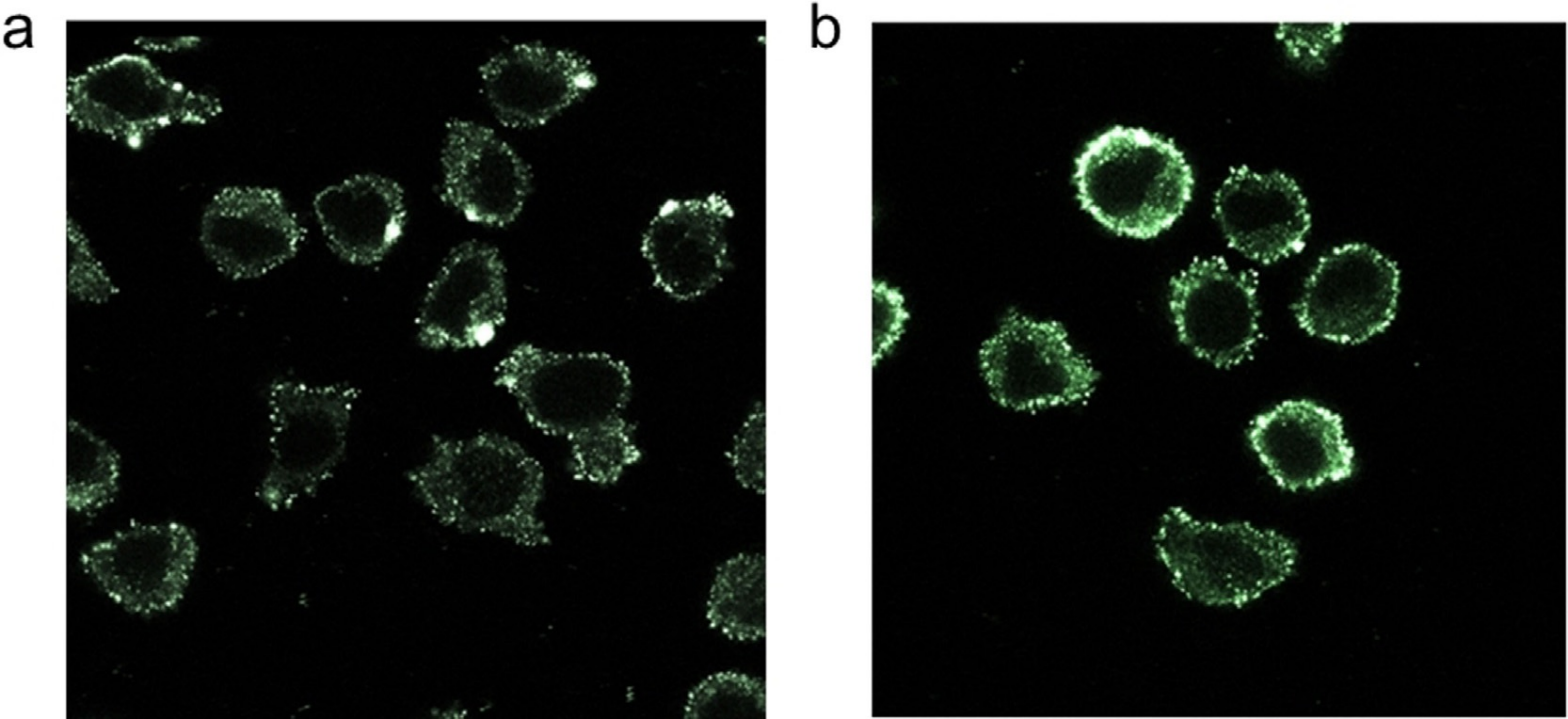
Confocal fluorescence microscopy experiment with M21 cell line after incubation with or without the ARM **15** and normal human serum (50 %). The binding was revealed with Alexa‐Fluor488‐coupled secondary anti‐human IgM antibody (1:400). a) Control experiment without ARM; b) Experiment performed with the ARM **15** (100 nm).

Although unspecific IgM binding can be observed with negative M21‐L cells, the visualization of the binding by confocal fluorescence microscopy confirmed the formation a complex between the M21 tumor cells, the ARM molecule **15,** and anti‐Rha IgM present in the human serum (Figure 6). It is also important to mention that the control compound **S5** displaying Gal instead of Rha (Figure S42 in the Supporting Information) did not show a positive signal, thus confirming that the formation of the ternary complex is specific to both the ABM and TBM and not to the peptide carrier itself.

## Conclusion

Immunotherapy represents one of the most promising way to treat cancer. If the utility of synthetic chemistry has been demonstrated with the development of active antitumoral vaccines, advanced clinical trials still remain unsatisfactory due to important hurdles, such as low molecular definition, reproducibility, and selectivity. Moreover, the need for efficient immunization strategies for cancer patients who generally suffer from immunodeficiency strongly hampers their development. The use of synthetic antibody recruiting molecules composed of ABM and TBM to guide natural antibodies already present in the blood stream to cancer cells, appears as a more suitable and direct approach because it does not require a pre‐immunization step. In this present work, we have first identified potent ABM and TBM molecules and demonstrated for the first time the importance of multivalent presentation of Rha and cRGD for efficient recognition with natural IgM and α_V_β_3_ integrin expressing M21 tumor cell line. Once covalently conjugated by click chemistry, we confirmed that recognition properties are conserved without pre‐immunization, and more importantly, that the ARM is able to promote the formation of a ternary complex between natural IgM and cancer cells, which is required for the stimulation of cytotoxic immune response in vivo.^5^, ^6^ These experiments are currently performed in our group and will be reported in due course.

## Experimental Section

### General procedure for the preparation of ARMs by CuAAC

Azido‐functionalized ABM (1 equiv) and alkyne‐functionalized TBM (1 equiv) were solubilized in a 1:1 mixture of DMF and PBS buffer (pH 7.5, 1 mL). A solution of CuSO_4_ **⋅**5 H_2_O (0.2 equiv) and THPTA (0.4 equiv) in PBS was added to a solution of sodium ascorbate (1 equiv) in PBS. This mixture was added to the solution containing the azide and alkyne solution, which was degassed with argon and stirred at RT for 2 h after which RP‐HPLC showed completion of the reaction. Chelex® resin was then added to the reaction mixture, which was stirred for 45 min. The resin was filtered off, rinsed with water, and the filtrate was purified by semi‐preparative RP‐HPLC. Fractions containing the product were combined and lyophilized.

### ABM binding assay

The ability of the ABMs to recruit anti‐Rha antibodies present in human serum (HS) was evaluated by an indirect competitive ELISA binding study. 96‐Well microtiter plates (Costar, flat bottom) were first coated with a solution of rhamnose‐functionalized poly[*N*‐52‐hydroxyethyl)acrylamide] polymer (PAA‐Rha, Lectinity) at 5 μg mL^−1^ in carbonate buffer 50 nm, pH 9.6 (100 μL/well, for 1 h at 37 °C). The wells were then washed with T‐PBS (3×100 μL per well, PBS pH 7.4 containing 0.05 % (v/v) Tween 20). This washing procedure was repeated after each incubation step. The coated microtiter plates were then blocked with bovine serum albumin (BSA) in PBS (3 % w/v, 1 h at 37 °C, 100 μL per well). Two serial dilutions of glycoconjugates were performed and incubated with HS 20 % (Sigma–Aldrich) then 100 μL of this mixture was added to the rhamnose‐coated wells. After 1 h of incubation at 37 °C, binding of the human anti‐Rha IgM present in HS to the immobilized rhamnose was revealed by Alexa‐Fluor488 goat anti‐human IgM secondary antibody (1:400, 100 μL per well, incubation 1 h at 37 °C, Fisher scientific). The fluorescence has been measured by using an Omega BMG LABTECH plate reader (Ex: 488 nm/ Em: 530 nm). The percentage of binding inhibition of the human anti‐Rha IgM antibody to the rhamnose‐coated microplate was calculated by using the formula: inhibition (%)=((*F*_max_−*F*)/*F*_max_)×100. *F*_max_ corresponds to the mean value of the higher fluorescence limit reached in the dilution series of each rhamnose conjugate whereas *F* is the fluorescence red for each concentration of the compound tested. The fluorescence values used in this formula were an average of triplicate experiments. The percent of inhibition versus the logarithm of the concentration for each compound (in mm) was plotted and the IC_50_ values were determined at 50 % of inhibition. The sigmoidal curves were fitted by using the Origin v6.1 software.

### Cell lines

Human M21 and M21‐L (modified to express low levels of α_v_β_3_ integrins) melanoma cell lines were kindly provided by J.L. Coll (IAB laboratory, Université Grenoble Alpes). Cells were cultured in Dulbecco's modified eagle medium (DMEM) supplemented with 10 % (v/v) of fetal bovine serum, 100 U mL^−1^ penicillin, and 100 μg mL^−1^ streptomycin (all were purchased from Sigma–Aldrich). Cells were maintained at 37 °C in a humidified atmosphere containing 5 % CO_2_. A solution of trypsin‐ethylenediaminetetraacetic acid (EDTA) at 0.05 % in DMEM (Sigma–Aldrich) was used for subculture to maintain cells in the exponential growth phase.

### TBM binding assay

Near confluent cells were harvested, washed, counted, and resuspended at a density of 1×10^6^ cells mL^−1^ in HBSS buffer (Hank's balanced salt solution purchased from Sigma–Aldrich) during 20 min at 4 °C. After a centrifugation step at 300 g for 5 min, cells were incubated with fluorescent FITC–TBMs conjugates **9** and **11** (5 μm) in DMEM at 37 °C for 1 h. Subsequently, the cells were centrifuged, wash once (HBSS), resuspended in 1 mL of HBSS and immediately analyzed by using a BD LSR FORTESSA flow cytometer (Becton Dickinson). The fluorophore was excited with a *λ*=488 nm laser (100 mW) and the fluorescence emission was collected with a 525/50 bypass filter. Data were analyzed by using the FCS express 6 software (De Novo Software).

### Binding assay with purified anti‐Rha antibody

Cells in suspension obtained as described previously (1×10^6^ cells mL^−1^ in HBSS) were fixed with paraformaldehyde (PFA) (4 % in PBS) during 10 min at 37 °C, followed by a neutralization on ice during 1 min. After two washes with HBSS, cells were incubated with ARMs **14** and **15** (100 nm in DMEM) for 1 h at room temperature. Purified rabbit antibodies selected against the molecule **2** (Davids Biotechnologie GmbH) were then added (10 μg mL^−1^ in DMEM) to the washed cells and the incubation was continued for 2 h at room temperature. After a second round of washing, binding of the anti‐Rha antibodies to the cells was revealed by using a PE‐conjugated goat anti‐rabbit secondary antibody (1:100 in DMEM, 1 h of incubation at room temperature, Fisher Scientific). Cells washed and resuspended in 1 mL of HBSS were immediately analyzed for PE intensity in the flow cytometer (BD LSR FORTESSA, laser excitation at *λ*=488 nm, bypass emission filter at 575/26) and by confocal microscopy (TCS SP8 CSU, Leica, laser excitation at *λ*=552 nm and fluorescence emission was collected between *λ*=650 and 740 nm).

### Human serum anti‐Rha antibody recruiting assay

Cells (1.5×10^6^ cells mL^−1^ in HBSS) were fixed with PFA (as described previously) then incubated with the ARMs **14** and **15** (100 nm in solution in DMEM) during 1 h at room temperature. After one wash with HBSS, cells were incubated with human serum (HS, 50 % in DMEM) obtained from a healthy human male donor (Sigma–Aldrich, H4522) for 2 h at room temperature. After one more washing, the anti‐Rha antibody binding was finally revealed by adding an Alexa‐Fluor488‐coupled anti‐human IgM secondary antibody (1:400). After 1 h of incubation at room temperature then washing (HBSS), cells were immediately analyzed for the Alexa‐Fluor488 intensity with a flow cytometer (BD LSR FORTESSA, laser excitation at *λ*=488 nm, emission bypass filter at 525/50) and a confocal microscope (laser excitation at *λ*=448 nm and fluorescence emission collected between *λ*=495 and 545 nm).

## Conflict of interest

The authors declare no conflict of interest.

## Supporting information

As a service to our authors and readers, this journal provides supporting information supplied by the authors. Such materials are peer reviewed and may be re‐organized for online delivery, but are not copy‐edited or typeset. Technical support issues arising from supporting information (other than missing files) should be addressed to the authors.

Supplementary
