# Supplementary material for: Multifunctional Glycoconjugates for Recruiting Natural Antibodies against Cancer Cells
Source: Chemistry. 2019 Oct 15;25(68):15508–15. doi: 10.1002/chem.201903327 (PMC6916168; doi:10.1002/chem.201903327)
Supplement: Supplementary file 1 — Supplementary [file CHEM-25-15508-s001.pdf]

CHEMISTRY
A European Journal

Supporting Information

# Multifunctional Glycoconjugates for Recruiting Natural Antibodies against Cancer Cells

Benjamin Liet, Eugénie Laigre, David Goyard, Biagio Todaro, Claire Tiertant, Didier Boturyn, Nathalie Berthet,* and Olivier Renaudet*[a]

chem_201903327_sm_miscellaneous_information.pdf

## Supporting Information

# Multifunctional glycoconjugates for recruiting natural antibodies against cancer cells

Benjamin Liet,[a] Eugénie Laigre,[a] David Goyard,[a] Biagio Todaro,[a] Claire Tiertant,[a] Didier Boturyn,[a] Nathalie Berthet*,[a] and Olivier Renaudet*,[a]

[a] Univ. Grenoble Alpes, CNRS, DCM UMR 5250, F-38000 Grenoble, France

*Corresponding author: olivier.renaudet@univ-grenoble-alpes.fr; nathalie.berthet@univ-grenoble-alpes.fr

General methods ........ 2
Synthetic procedures ........ 2
NMR spectra of final compounds ........ 23
ELISA assay ........ 27
Confocal microscopy analysis of M21 cells incubated with 9 and 11 ........ 27
Cells αvβ3 integrins quantification ........ 27
Binding of human serum against galactosylated control ........ 28
References ........ 29

## General methods

All chemical reagents were purchased from Aldrich (Saint Quentin Fallavier, France) or Acros (Noisy-Le-Grand, France) and were used without further purification. All protected amino acids and Fmoc-Gly-Sasrin®resin was obtained from Advanced ChemTech Europe (Brussels, Belgium), BachemBiochimie SARL (Voisins-Les-Bretonneux, France) and France Biochem S.A. (Meudon, France). For peptides and glycopeptides, analytical RP-HPLC was performed on a Waters alliance 2695 separation module, equipped with a Waters 2489 UV/visible detector. Analyses were carried out at 1.23 mL/min (Interchim UPTISPHERE XSERIE, $C_{18}$, 5 µm, 125x3.0 mm) with UV monitoring at 214 nm and 250 nm using a linear A–B gradient (buffer A: 0.09% $CF_3CO_2H$ in water; buffer B: 0.09% $CF_3CO_2H$ in 90% acetonitrile). Preparative HPLC was performed on Gilson GX 281 equipped with a fraction collector or on Waters equipment consisting of a Waters 600 controller and a Waters 2487 Dual Absorbance Detector. Purifications were carried out at 22.0 mL/min (VP 250x21 mm nucleosil 100-7 $C_{18}$) with UV monitoring at 214 nm and 250 nm using a linear A–B gradient. $^1$H spectra were recorded on BrukerAvance III 500 MHz spectrometers and chemical shifts ($\delta$) were reported in parts per million (ppm). Spectra were referenced to the residual proton solvent peaks relative to the signal of $D_2O$ (4.79 ppm for $^1$H). ESI mass spectra of peptides and glycopeptides were measured on an Esquire 3000 spectrometer from Bruker. MALDI-ToF were performed on a AutoFlex I Bruker after sample pretreatment in an OligoR3 microcolumn (Applied Biosystems, USA) using 2,5-dihydroxybenzoic acid matrix. HRMS analyses were performed on a Waters Xevo® G2-S QTof at Mass Spectrometry facility, PCN-ICMG, Grenoble.

## Synthetic procedures

General procedure **A** for the preparation of glycoclusters and glycodendrimers by CuAAC.

Propargyl glycoside or alkyne-substituted glycocluster (4.4 eq.) and azide-functionalized scaffold (1 eq.) were dissolved in 1 mL of a 1:1 mixture of DMF and PBS buffer (pH 7.5). A solution of $CuSO_4 \cdot 5H_2O$ (0.5 eq.) and THPTA (1 eq.) in PBS was added to a solution of sodium ascorbate (3 eq.) in PBS. This mixture was added to the solution containing the azide and alkyne which was degassed with argon and stirred at r.t. for 2 hours after which RP-HPLC showed completion of the reaction. Chelex® resin was then added to the reaction mixture which was stirred for 45 minutes. The resin was filtered off, rinsed with water and the filtrate purified by semi-preparative RP-HPLC. Fractions containing the product were combined and lyophilized.

General procedure **B** for the introduction of alkyne and azide on the free lysine residue of multivalent constructions

Tetra- or hexadecavalent compound (1 eq.) was dissolved in dry DMF (1 mL), DIPEA was added to reach pH ~ 9-10 (c.a. 20 µL) then succinimide ester of pentynoic or azidoacetic acid (1.5 eq.) was added. The reaction mixture was stirred at r.t. for 1 hour after which RP-HPLC showed completion of the reaction. The mixture was diluted with

water (3 mL) and purified by semi-preparative RP-HPLC. Fractions containing the product were combined and lyophilized.

General procedure **C** for the preparation of ARM's by CuAAC

Azido-functionalized ABM (1eq.) and alkyne-functionalized TBM (1 eq.) were solubilized in 1 mL of a 1:1 mixture of DMF and PBS buffer (pH 7.5). A solution of $CuSO_4{\cdot}5H_2O$ (0.2 eq.) and THPTA (0.4 eq.) in PBS was added to a solution of sodium ascorbate (1 eq.) in PBS. This mixture was added to the solution containing the azide and alkyne which was degassed with argon and stirred at r.t. for 2 hours after which RP-HPLC showed completion of the reaction. Chelex® resin was then added to the reaction mixture which was stirred for 45 minutes. The resin was filtered off, rinsed with water and the filtrate purified by semi-preparative RP-HPLC. Fractions containing the product were combined and lyophilized.

Compound **2**

Prepared according general procedure **A** from propargyl α-L-Rhamnopyranoside (11.5 mg, 57 µmol) and **1** (14.5 mg, 13 µmol). The crude mixture was purified to afford the title compound as a white fluffy solid after lyophilization (20.5 mg, 11 µmol, 82%). HRMS (ESI$^+$-TOF) *m/z*: calcd for $C_{83}H_{134}N_{23}O_{30}$ $[M+H]^+$: 1932.9667, found 1932.9700; RP-HPLC: $R_t$ = 9.66 min (C18, λ = 214 nm, 0-30% B in 15 min).

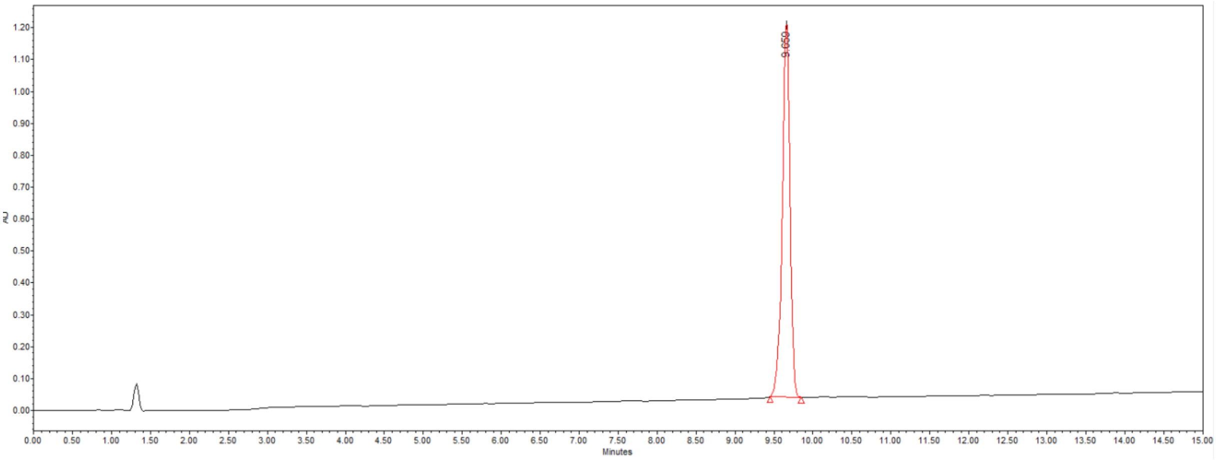


Figure S 1. RP-HPLC Spectrum of compound **2**

| Target | Mass Found | Error PPM | Compound |
|---|---|---|---|
| 1932.9667 | 1932.97 | 1.7 | Found |

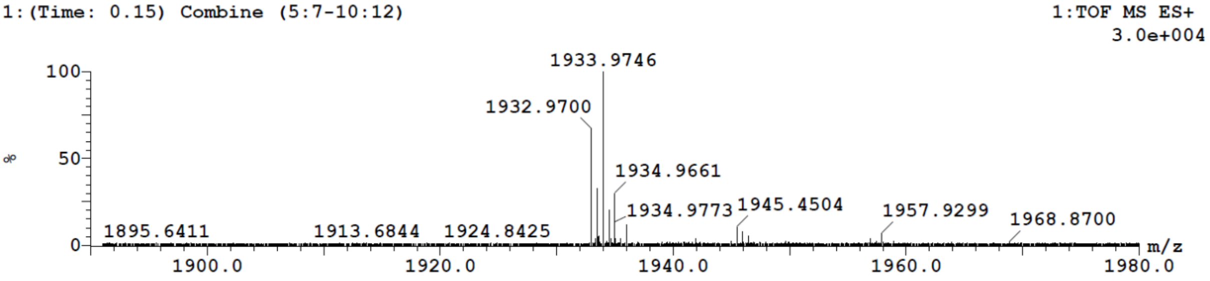


| Mass | PPM | i-FIT (norm) | Formula | Calc. Mass |
|---|---|---|---|---|
| 1932.9700 | 1.7 | 1.9 | C83 H134 N23 O30 | 1932.9667 |

Figure S 2. HRMS spectrum of compound **2**

Compound **3**

Prepared according general procedure **B** from **2** (32.0 mg, 16.6 µmol) and azidoacetic acid succinimide ester (4.9 mg, 24.8 µmol). The crude mixture was purified to afford the title compound as a white fluffy solid after lyophilization (30.7 mg, 15.2 µmol, 92%). HRMS (ESI$^+$-TOF) *m/z*: calcd for $C_{85}H_{136}N_{26}O_{31}$ [M+2H]$^{2+}$: 1008.4927, found 1008.4916; RP-HPLC: $R_t$ = 9.15 min (C18, λ = 214 nm, 0-40% B in 15 min).

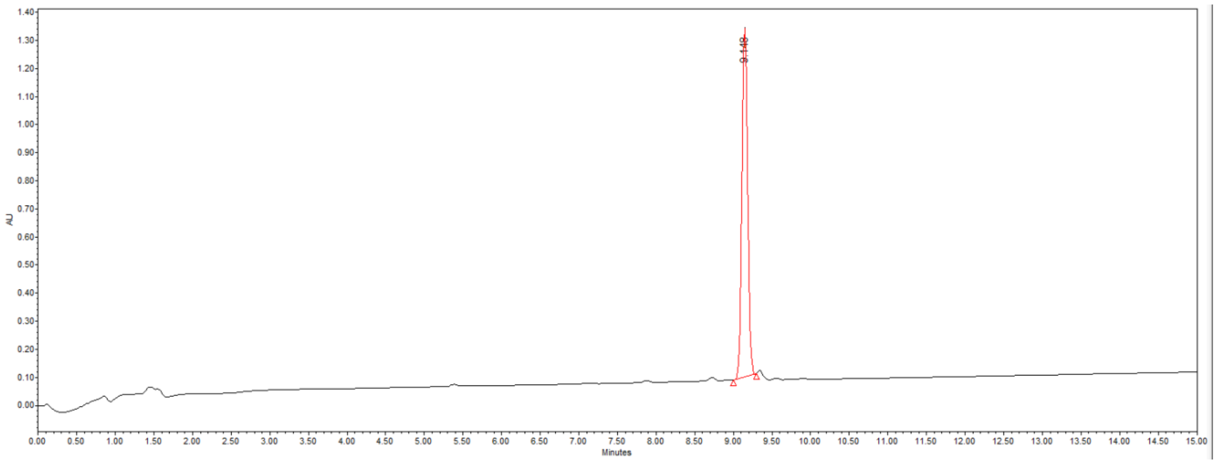


Figure S 3. RP-HPLC Spectrum of compound **3**

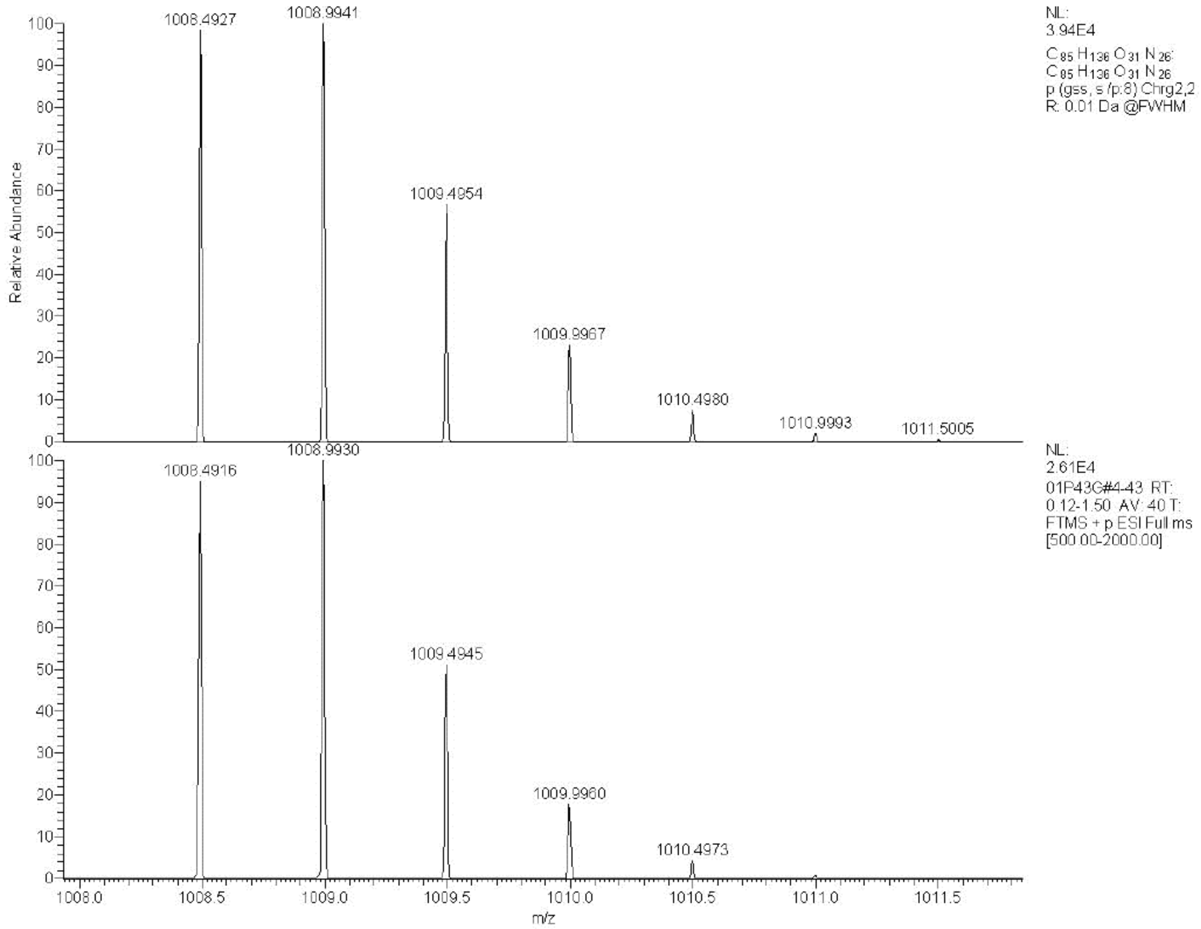


Figure S 4. HRMS spectrum of compound **3**

Compound **4**

Prepared according general procedure **B** from **2** (27 mg, 14.0 µmol) and pentynoic acid succinimide ester (4 mg, 20.9 µmol). The crude mixture was purified to afford the title compound as a white fluffy solid after lyophilization (25 mg, 12.4 µmol, 89%). HRMS (ESI$^+$-TOF) *m/z*: calcd for $C_{83}H_{134}N_{23}NaO_{30}$ [M+Na]$^+$: 2034.9749, found 2034.9777; RP-HPLC: $R_t$ = 9.30 min (C18, λ = 214 nm, 0-40% B in 15 min).

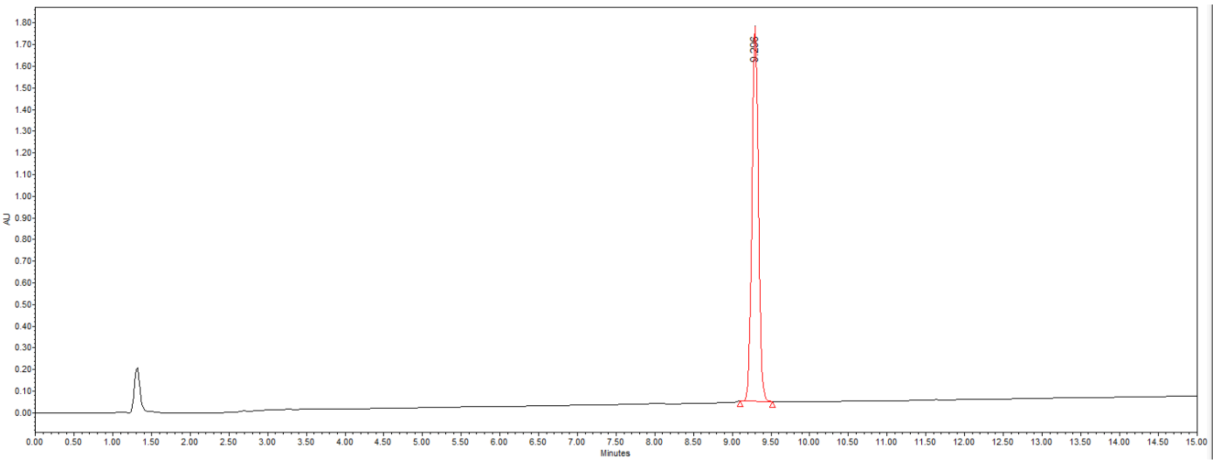


Figure S 5. RP-HPLC Spectrum of compound **4**

| Target | Mass Found | Error PPM | Compound |
|---|---|---|---|
| 2034.9749 | 2034.97 | 1.4 | Found |

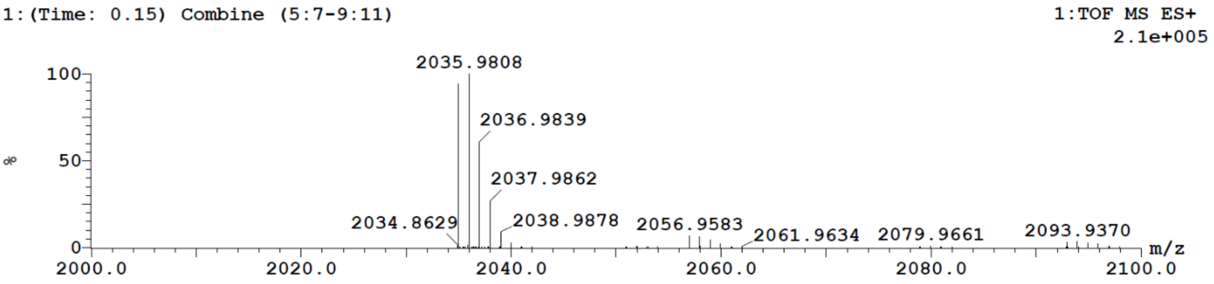


| Mass | PPM | i-FIT (norm) | Formula | Calc. Mass |
|---|---|---|---|---|
| 2034.9777 | 1.4 | 0.4 | C88 H137 N23 O31 Na | 2034.9749 |

Figure S 6. HRMS spectrum of compound **4**

## Compound **5**

Prepared according general procedure **A** from **4** (25.0 mg, 12.1 µmol) and **1** (3.0 mg, 2.7 µmol). The crude mixture was purified to afford the title compound as a white fluffy solid after lyophilization (18.6 mg, 2.0 µmol, 74%). HRMS (ESI$^+$-TOF) *m/z*: calcd for $C_{399}H_{625}N_{115}O_{134}$ [M+5H]$^{5+}$: 1835.3198, found 1835.3266; RP-HPLC: $R_t$ = 10.16 min (C18, λ = 214 nm, 0-40% B in 15 min).

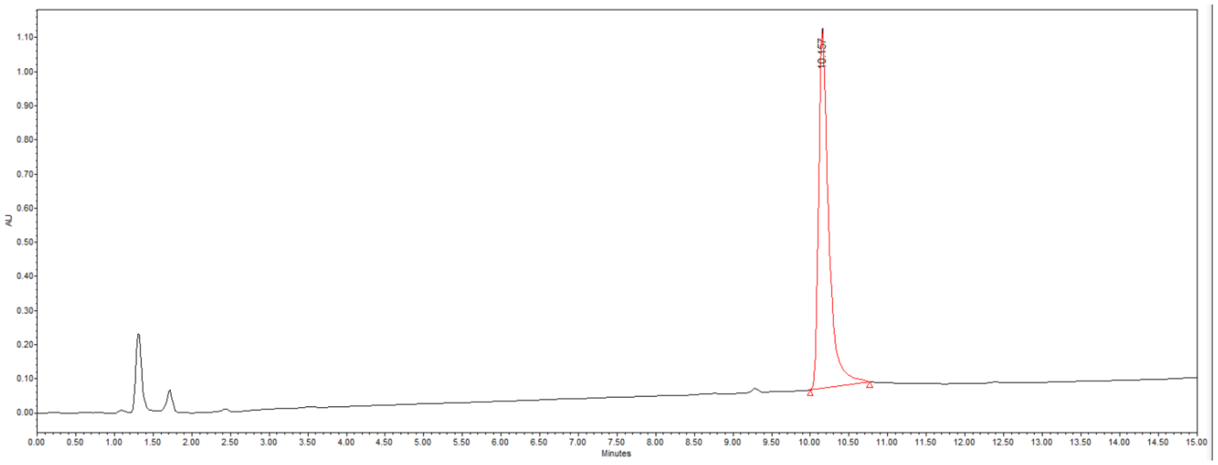


Figure S 7. RP-HPLC Spectrum of compound **5**

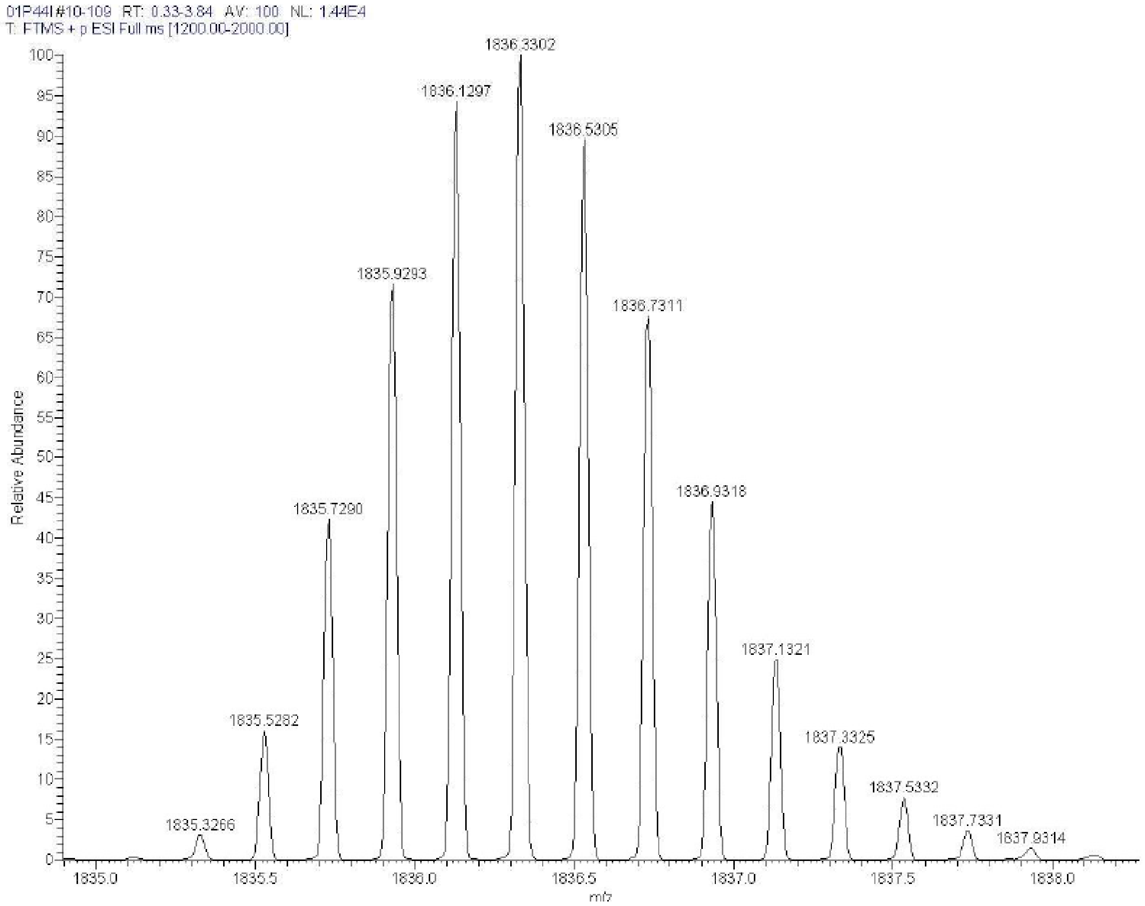

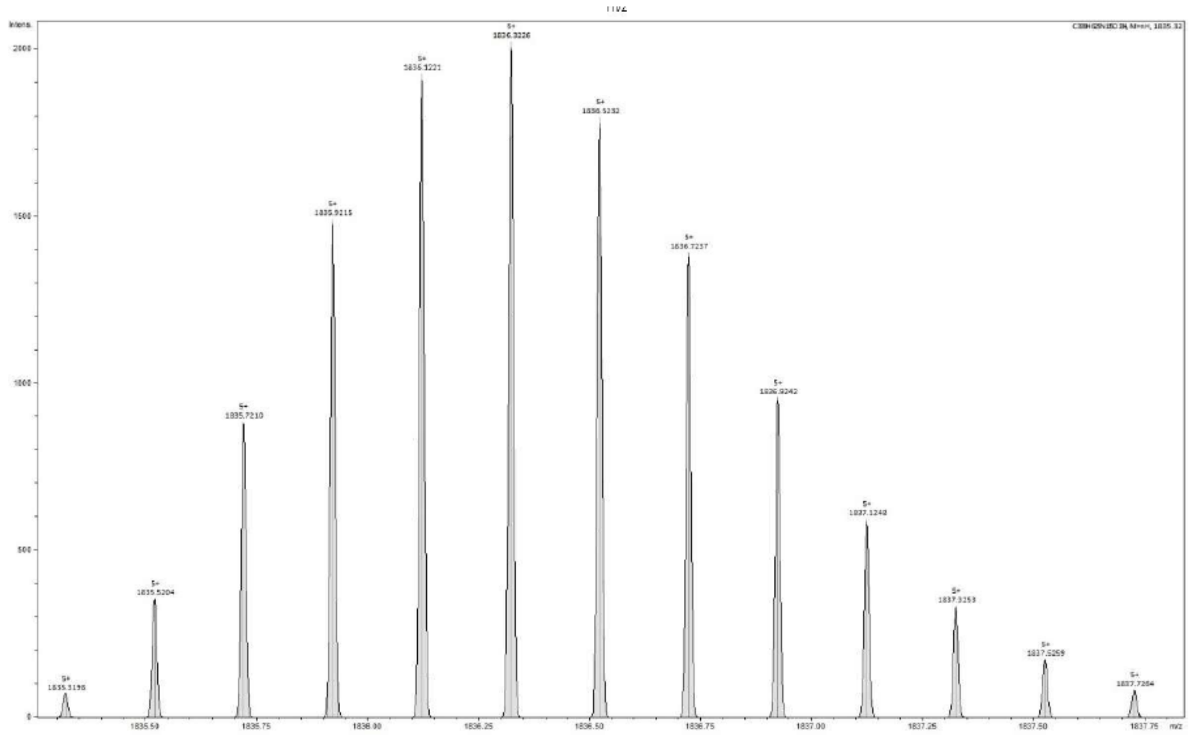

Figure S 8. HRMS spectrum of compound **5**

## Compound **6**

Prepared according general procedure **B** from **5** (24 mg, 2.6 µmol) and azidoacetic acid succinimide ester (0.8 mg, 3.9 µmol). The crude mixture was purified to afford the title compound as a white fluffy solid after lyophilization (18.4 mg, 2.0 µmol, 77%). MALDI-ToF *m/z*: calcd for $C_{401}H_{627}N_{118}O_{135}$ $[M+H]^+$: 9254.6, found 9255.0; RP-HPLC: $R_t$ = 4.97 min (C18, λ = 214 nm, 5-100% B in 15 min).

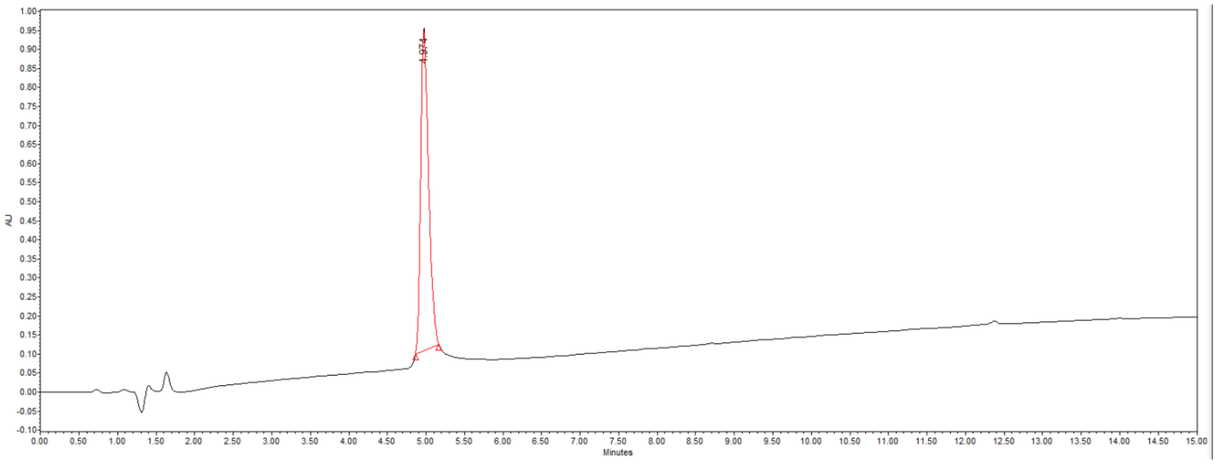

Figure S 9. RP-HPLC Spectrum of compound **6**

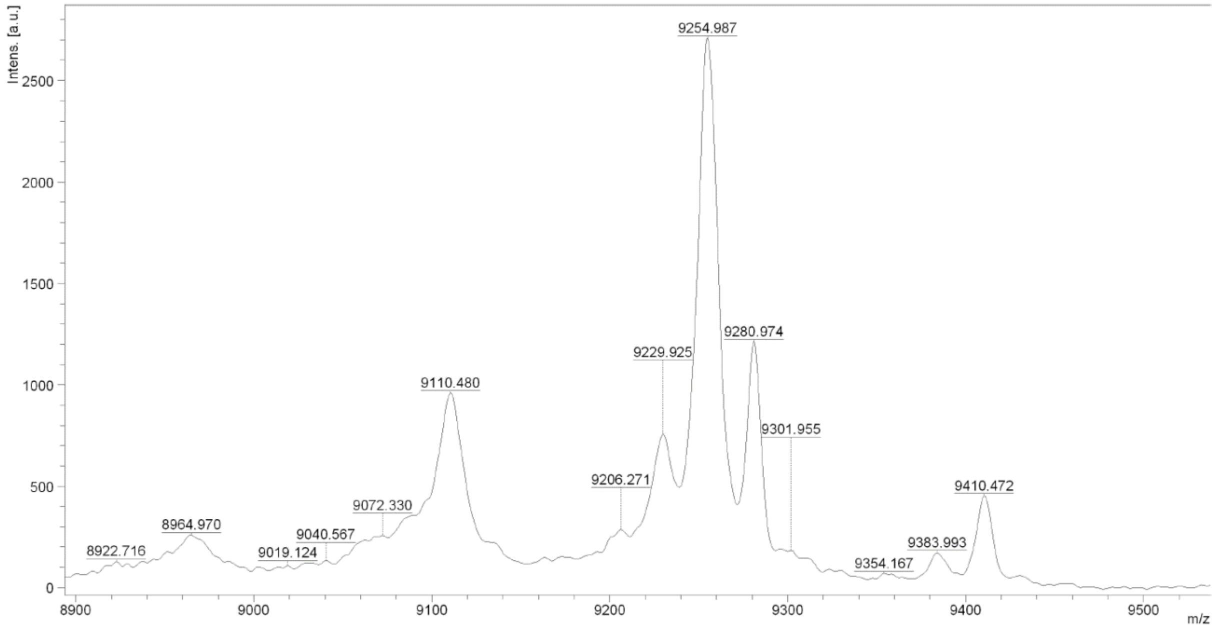


Figure S 10. MALDI-ToF spectrum of compound **6**

Compound **9**

Prepared according general procedure **C** from **7** (13 mg, 18.8 µmol) and **8** (13 mg, 18.8 µmol). The crude mixture was purified to afford the title compound as a yellow fluffy solid after lyophilization (16.8 mg, 12.2 µmol, 65%). HRMS (ESI$^+$-TOF) *m/z*: calcd for $C_{65}H_{82}N_{15}O_{17}S$ $[M+H]^+$: 1376.5728, found 1376.5764; RP-HPLC: $R_t$ = 6.88 min (C18, λ = 214 nm, 5-100% B in 15 min).

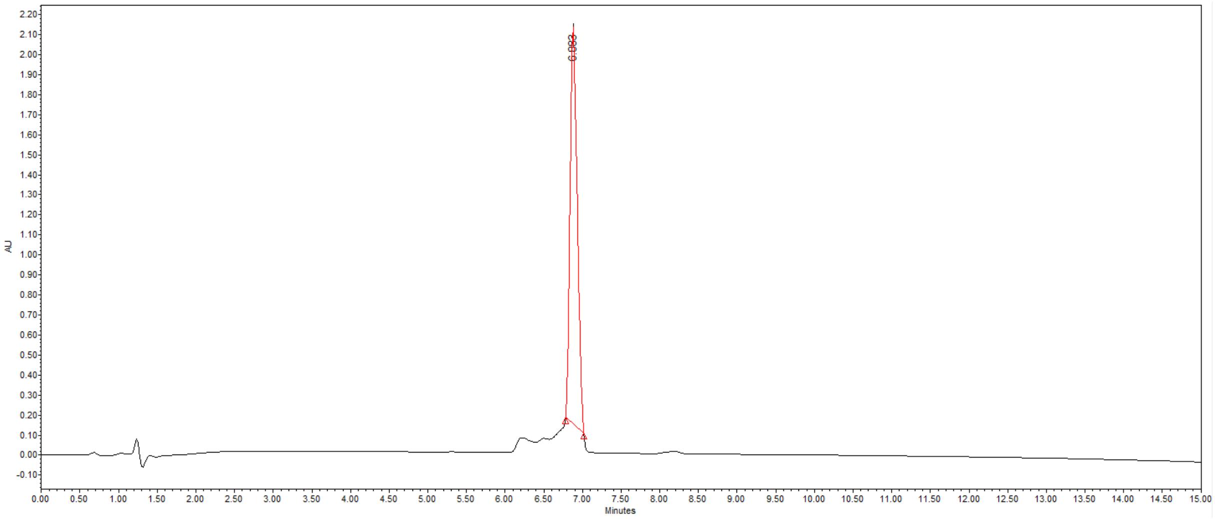


Figure S 11. RP-HPLC Spectrum of compound **9**

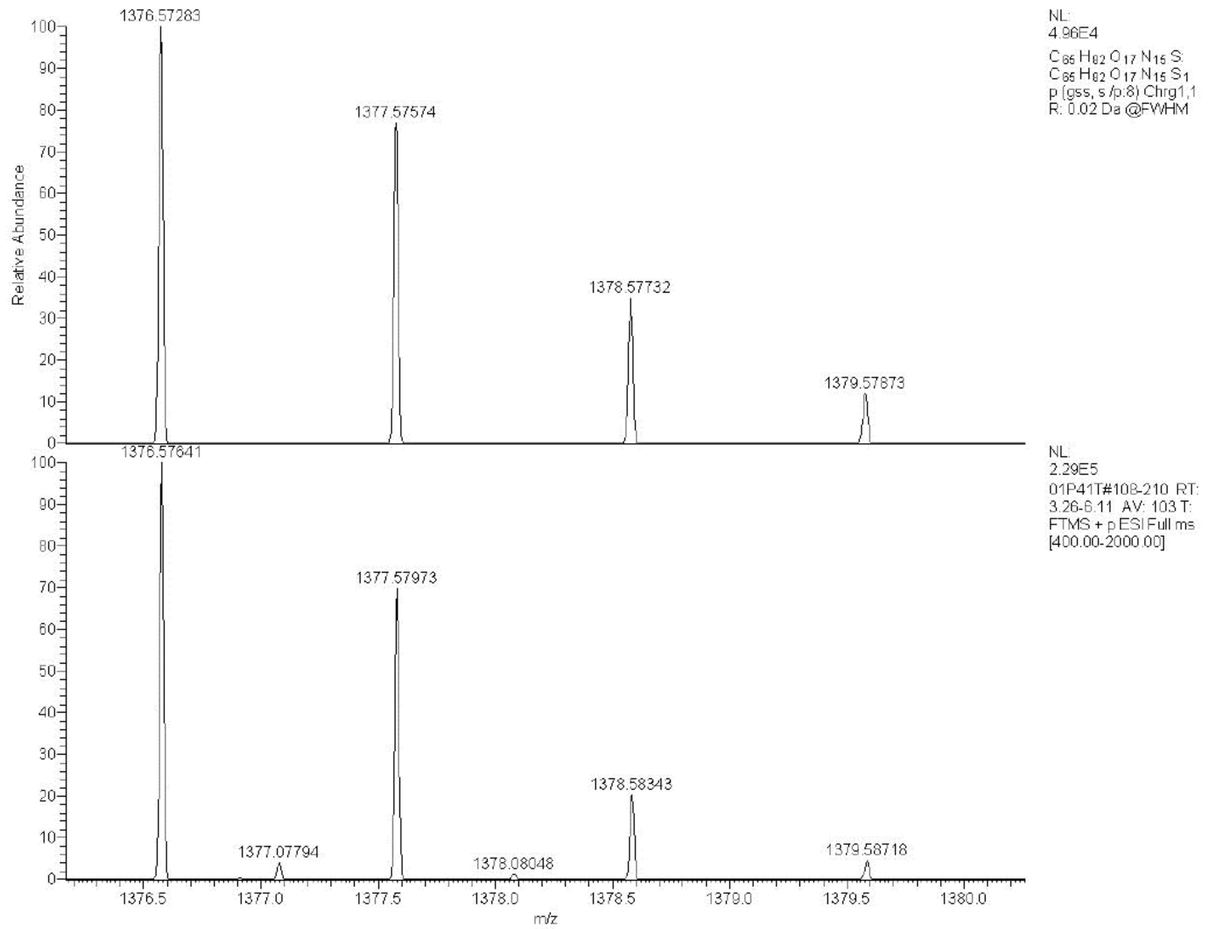


Figure S 12. HRMS spectrum of compound **9**

## Compound **11**

Prepared according general procedure **C** from **7** (2 mg, µmol) and **10** (11 mg, 2.8 µmol). The crude mixture was purified to afford the title compound as a yellow fluffy solid after lyophilization (10.2 mg, 2.2 µmol, 79%). HRMS (ESI$^+$-TOF) *m/z*: calcd for $C_{213}H_{300}N_{65}O_{52}S$ $[M+3H]^{3+}$: 1544.0844, found 1544.0894; RP-HPLC: $R_t$ = 6.06 and 6.40 min (C18, λ = 214 nm, 5-100% B in 15 min).

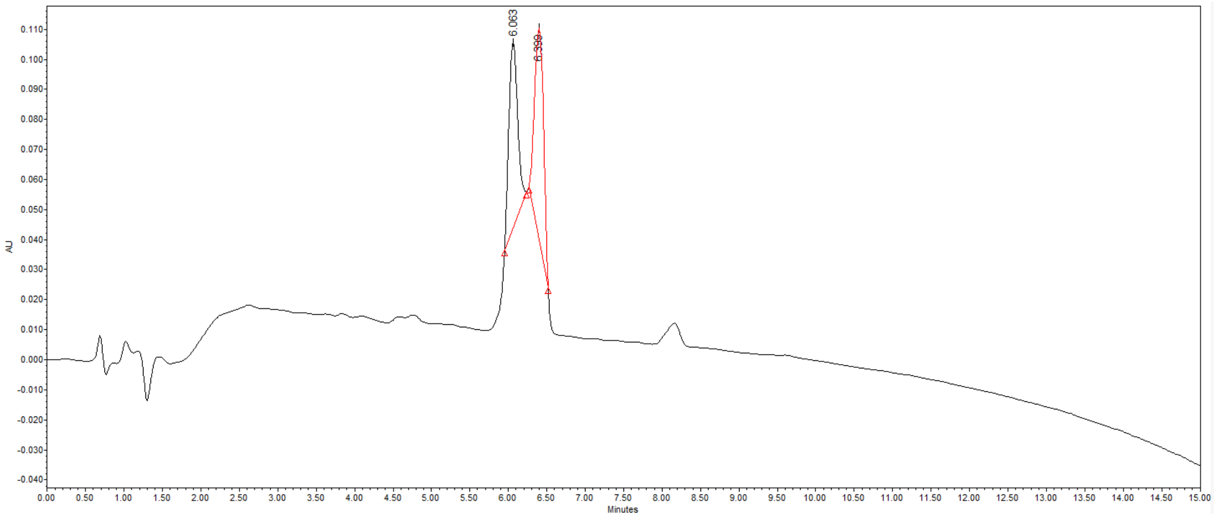


Figure S 13. RP-HPLC Spectrum of compound **11**

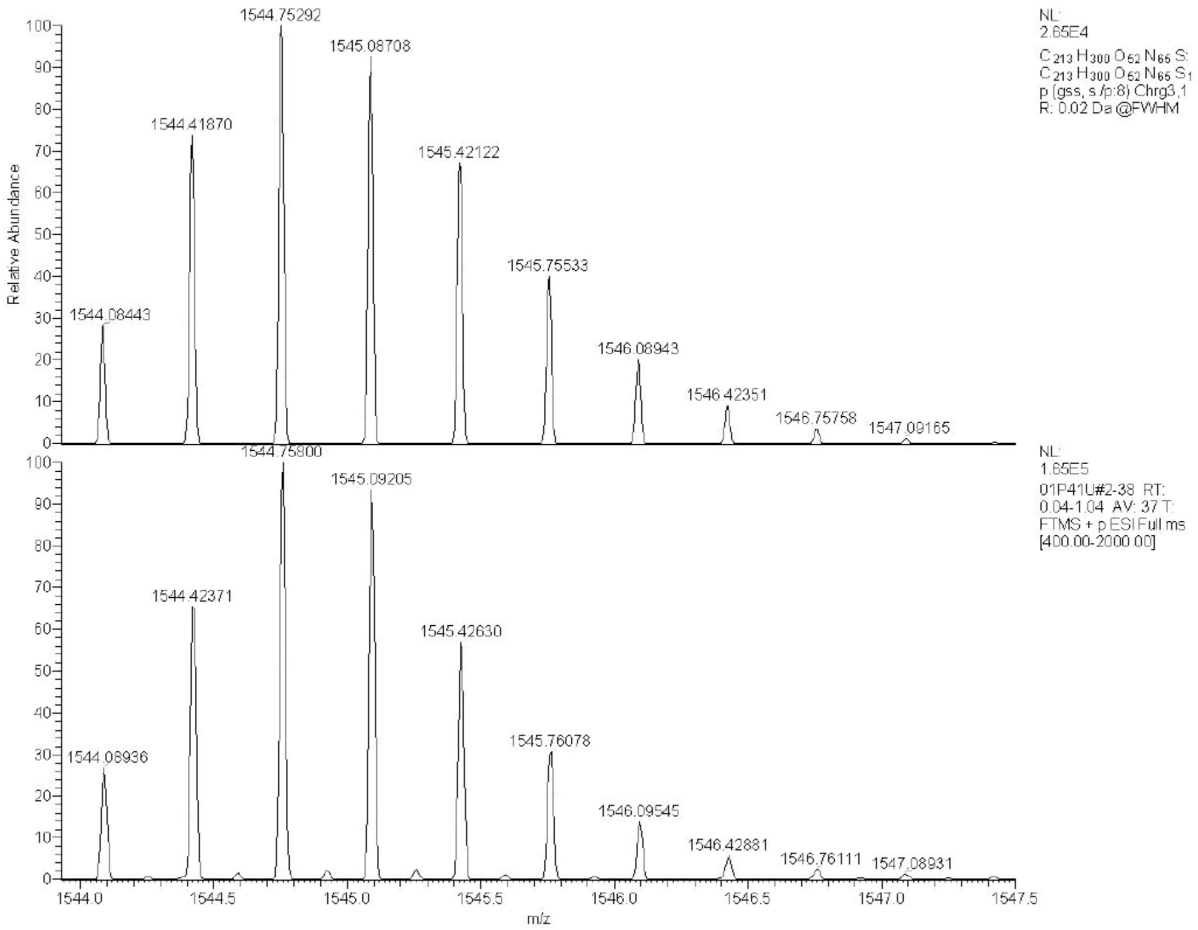


Figure S 14. HRMS spectrum of compound **11**

Compound **12**

Prepared according already described procedure. Analysis were in agreement with the literature.[1]

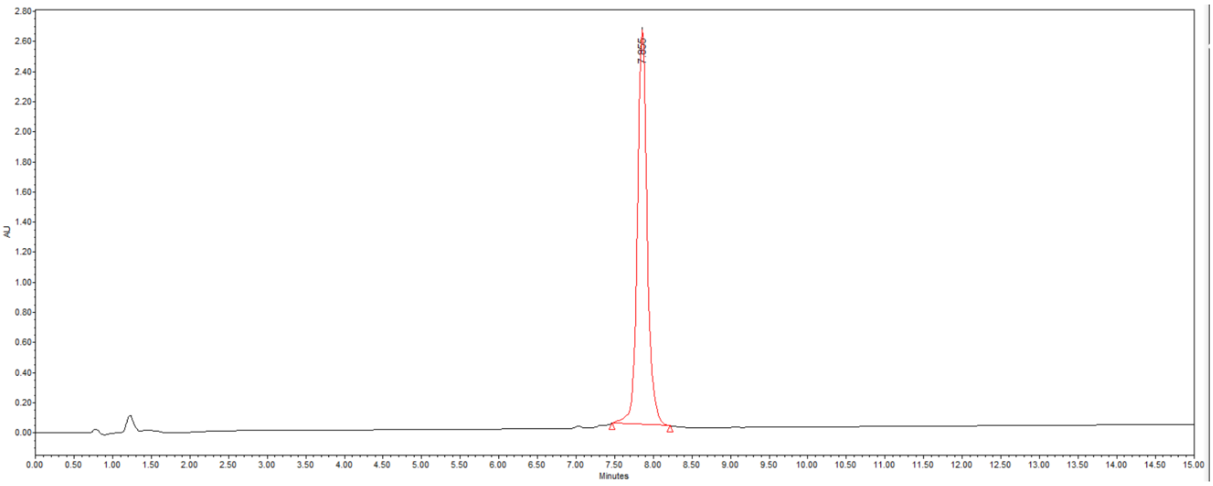


Figure S 15. RP-HPLC Spectrum of compound **12**

Compound **13**

Prepared according general procedure **B** from **12** (31.0 mg, 8.0 µmol) and pentynoic acid succinimide ester (2.4 mg, 12.1 µmol). The crude mixture was purified to afford the title compound as a white fluffy solid after lyophilization (21.8 mg, 5.5 µmol, 69%).

MALDI-ToF *m/z*: calcd for $C_{180}H_{262}N_{59}O_{43}$ $[M+H]^+$: 3940.4, found 3940.6; RP-HPLC: $R_t$ = 9.05 min (C18, λ = 214 nm, 0-60% B in 15 min).

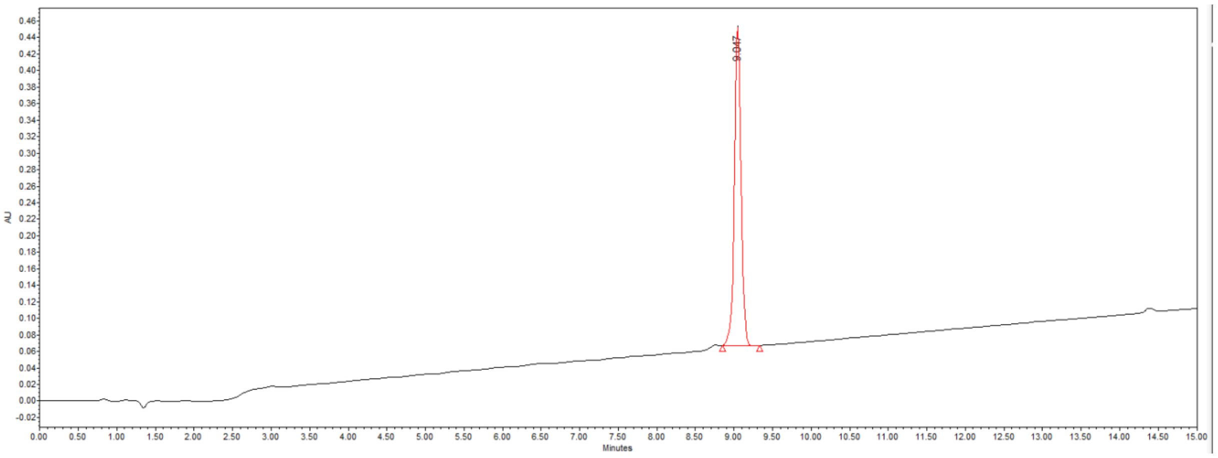


Figure S 16. RP-HPLC Spectrum of compound **13**

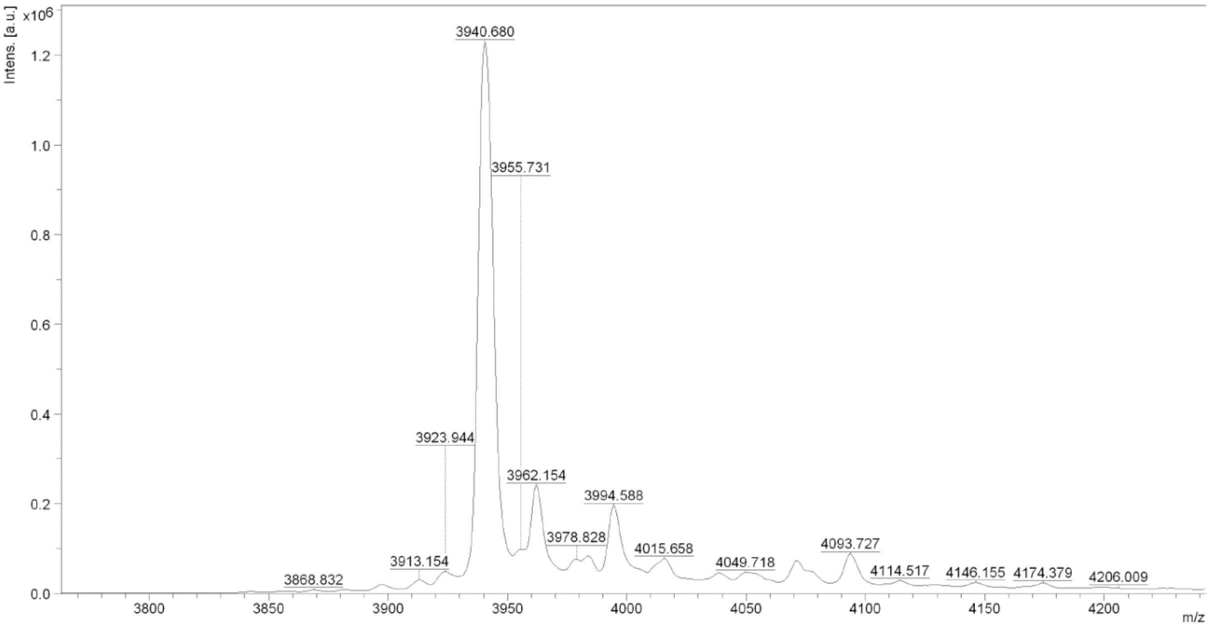


Figure S 17. MALDI-ToF spectrum of compound **13**

## Compound **14**

Prepared according general procedure **C** from **4** (5.1 mg, 2.5 µmol) and **13** (9.8 mg, 2.5 µmol). The crude mixture was purified to afford the title compound as a white fluffy solid after lyophilization (12.9 mg, 2.2 µmol, 86%). HRMS ($ESI^+$-TOF) *m/z*: calcd for $C_{265}H_{398}N_{85}O_{74}$ $[M+3H]^{3+}$: 1984.9992, found 1985.0065; RP-HPLC: $R_t$ = 7.72 min (C18, λ = 214 nm, 5-60% B in 15 min).

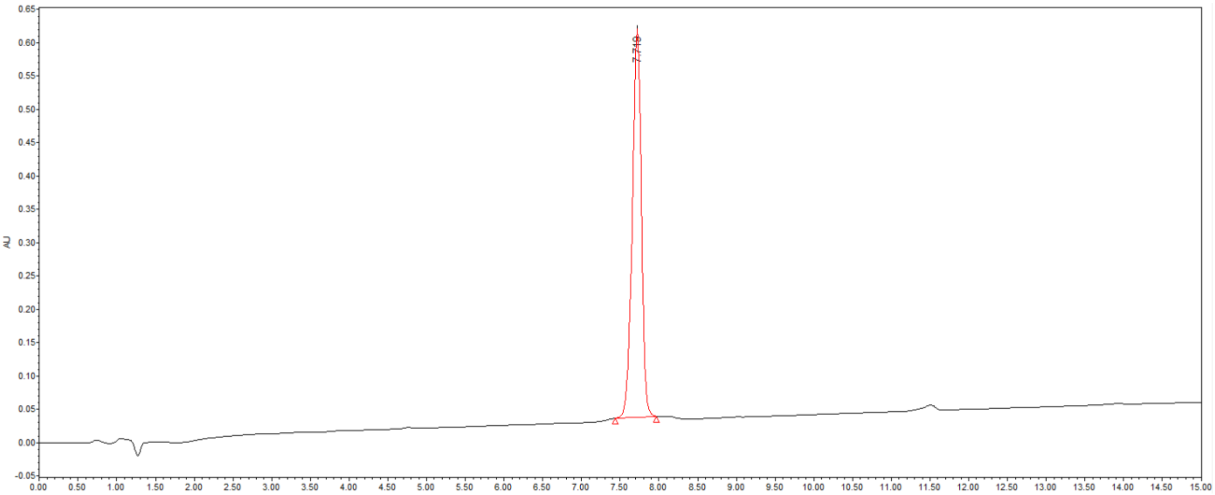


Figure S 18. RP-HPLC Spectrum of compound **14**

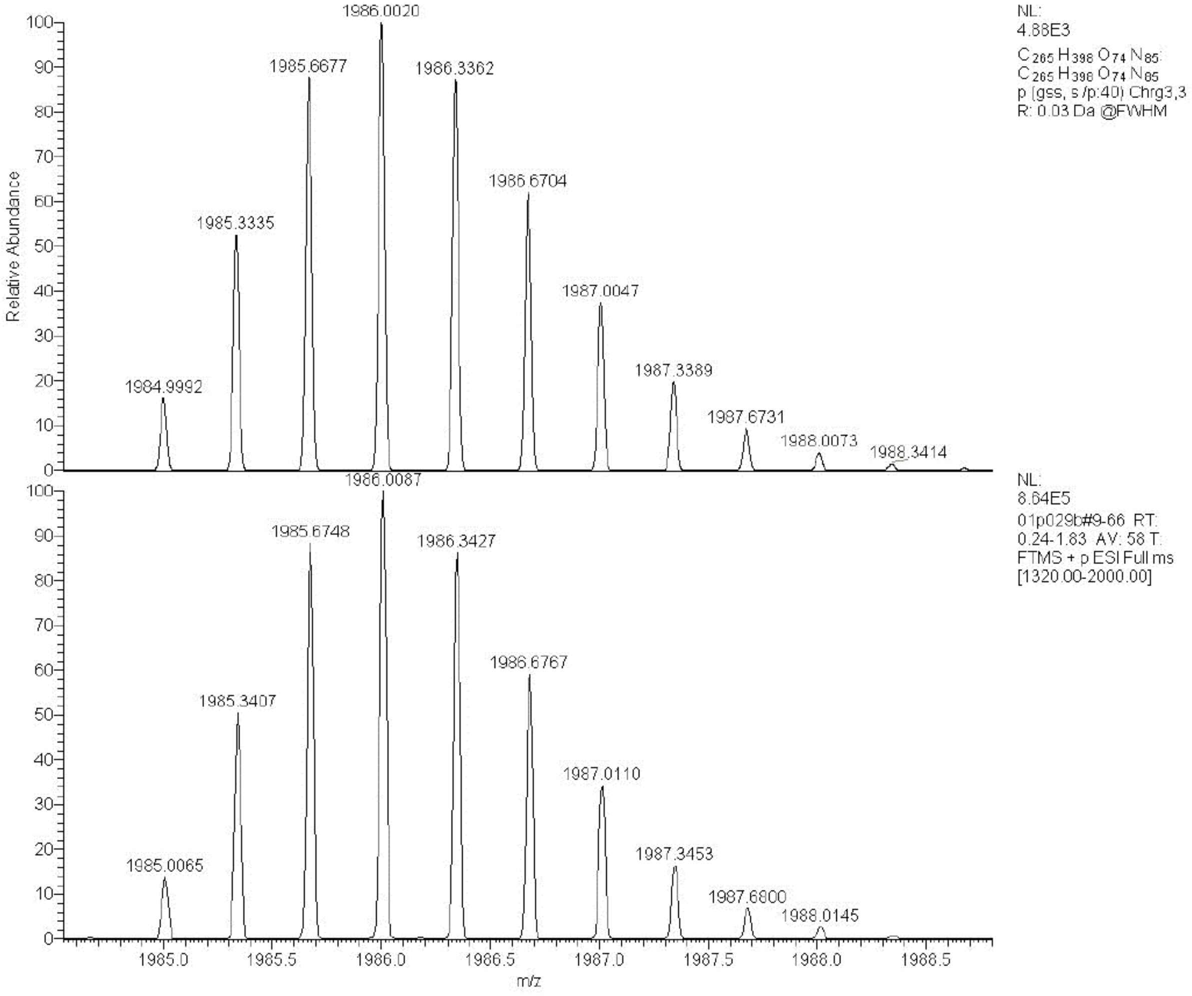


Figure S 19. HRMS spectrum of compound **14**

## Compound **15**

Prepared according general procedure **C** from **6** (7.2 mg, 0.78 µmol) and **13** (3.1 mg, 0.78 µmol). The crude mixture was purified to afford the title compound as a white fluffy solid after lyophilization (12.9 mg, 0.60 µmol, 78%). HRMS (ESI$^+$-TOF) *m/z*: calcd for $C_{581}H_{887}N_{177}O_{178}$ [M+7H]$^{7+}$: 1885.5187, found 1885.5321; RP-HPLC: R$_t$ = 7.16 min (C18, λ = 214 nm, 5-60% B in 15 min).

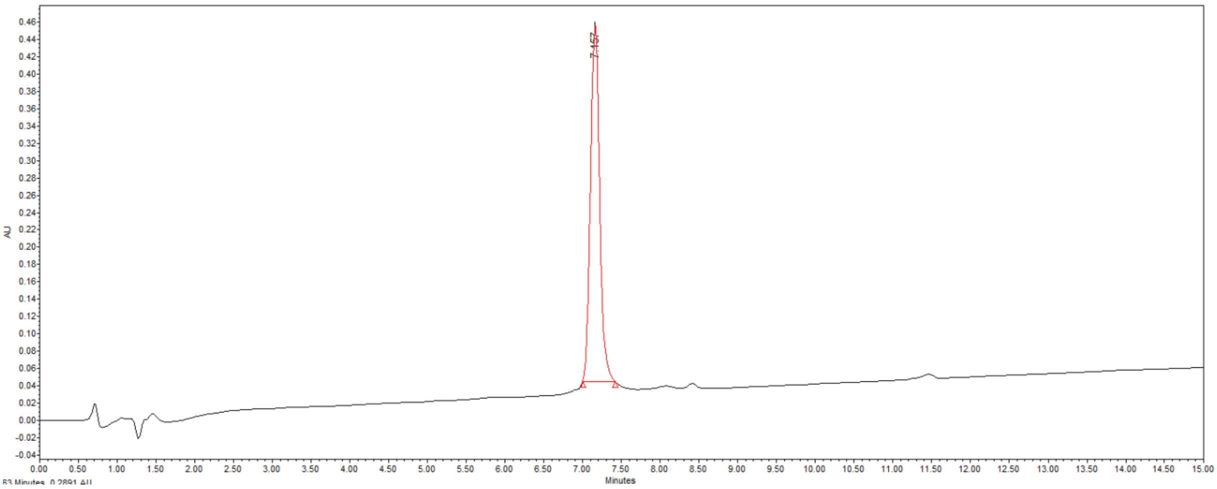


Figure S 20. RP-HPLC Spectrum of compound **15**

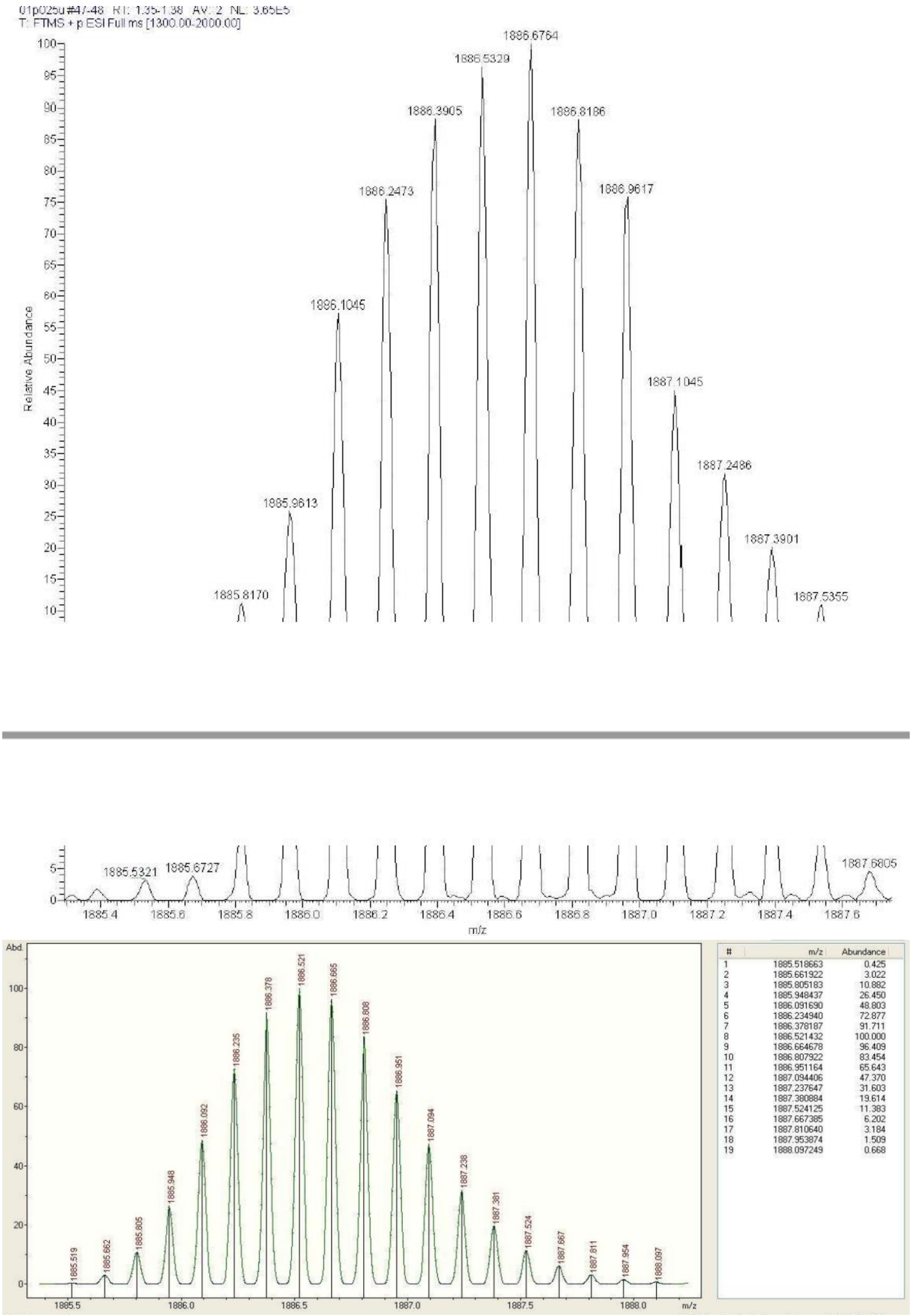


Figure S 21. HRMS spectrum of compound **15**

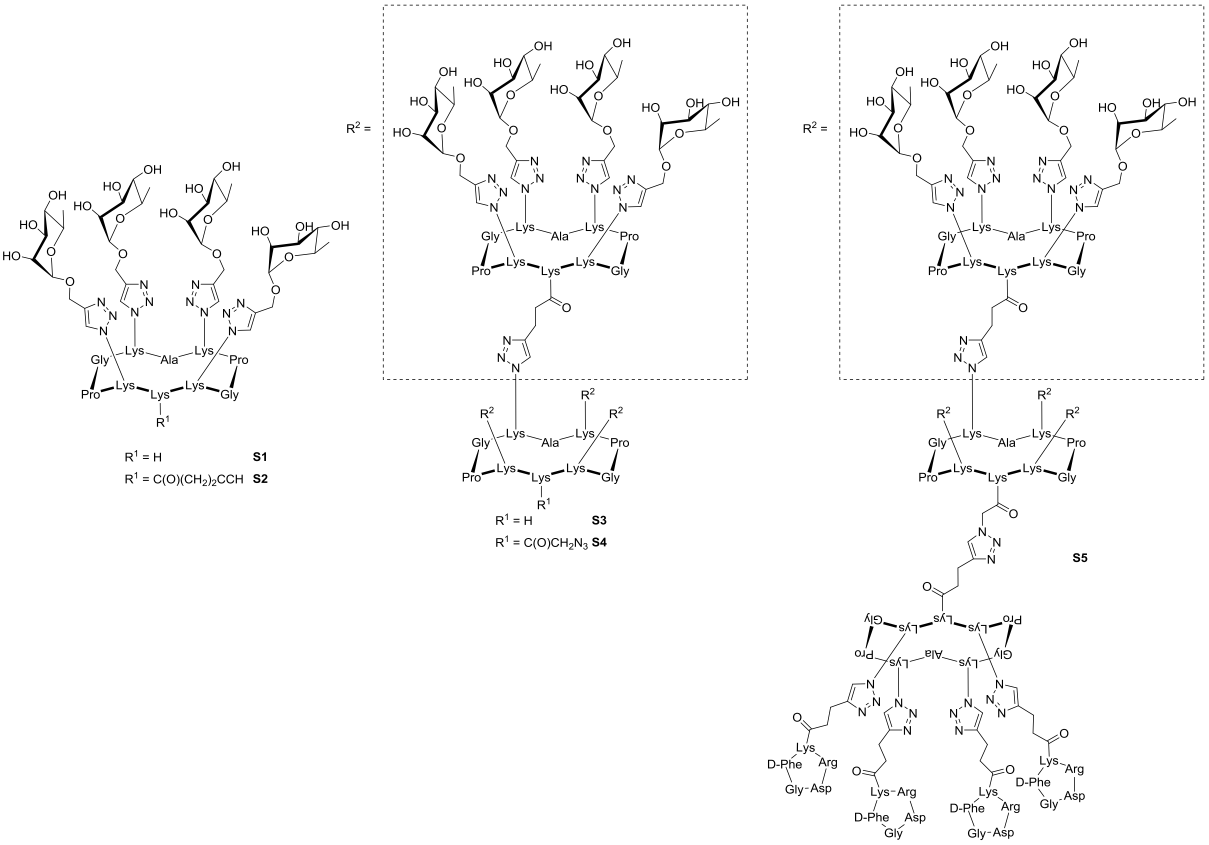


Compound **S1**

Prepared according general procedure **A** from propargyl β-D-galactopyranoside (27 mg, 124 µmol) and **1** (31.6 mg, 28.1 µmol). The crude mixture was purified to afford the title compound as a white fluffy solid after lyophilization (47.8 mg, 23.9 µmol, 85%). HRMS (ESI$^+$-TOF) *m/z*: calcd for $C_{83}H_{135}N_{23}O_{34}$ $[M+2H]^{2+}$: 998.9765, found 998.9779; RP-HPLC: $R_t$ = 8.81 min (C18, λ = 214 nm, 0-20% B in 15 min).

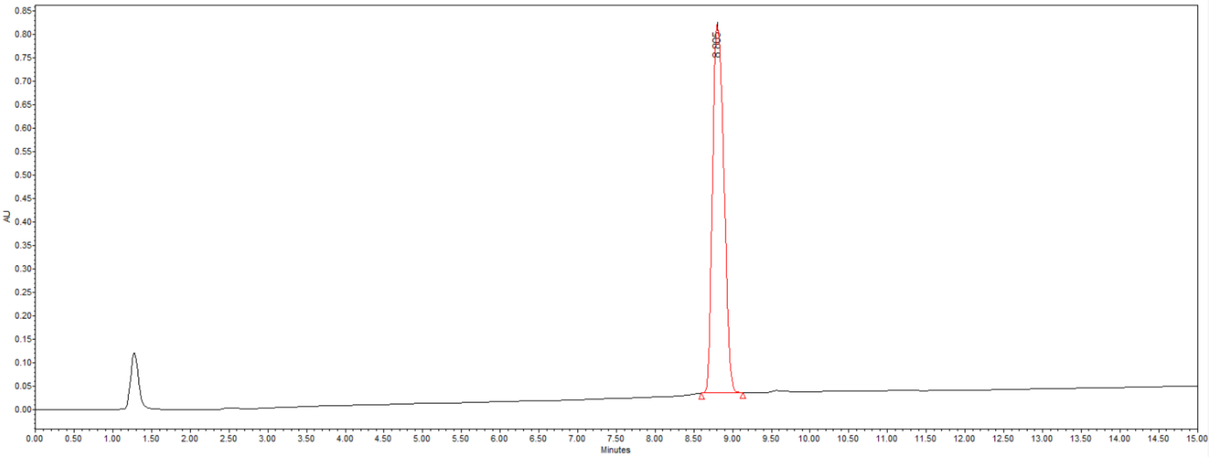


Figure S 22. RP-HPLC Spectrum of compound **S1**

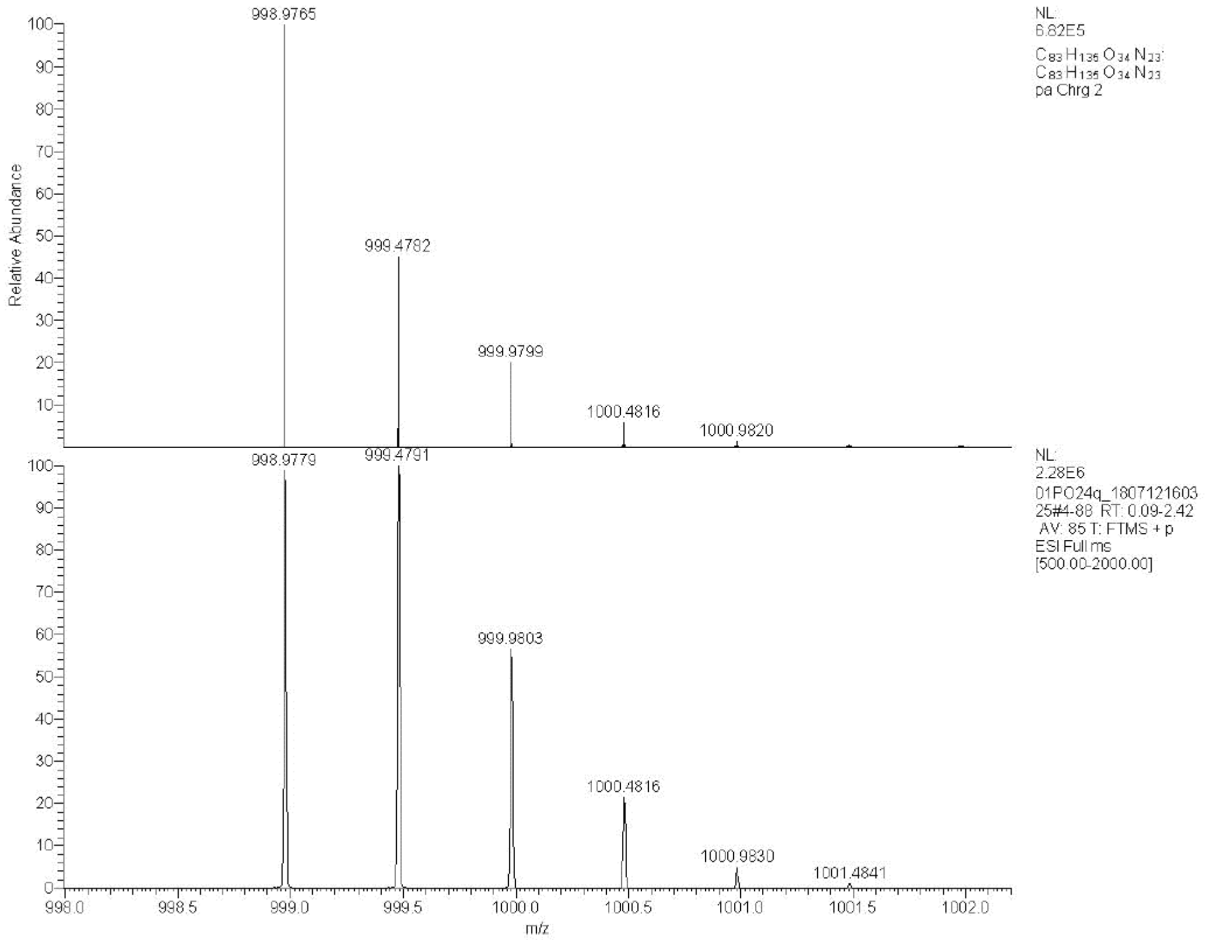


Figure S 23. HRMS spectrum of compound **S1**

## Compound **S2**

Prepared according general procedure **B** from **S1** (13.2 mg, 6.61 µmol) and pentynoic acid succinimide ester (1.9 mg, 9.92 µmol). The crude mixture was purified to afford the title compound as a white fluffy solid after lyophilization (12.4 mg, 5.95 µmol, 90%). HRMS (ESI$^+$-TOF) *m/z*: calcd for $C_{88}H_{139}N_{23}O_{35}$ [M+2H]$^{2+}$: 1038.9896, found 1038.9937; RP-HPLC: $R_t$ = 5.80 min (C18, λ = 214 nm, 5-40% B in 15 min).

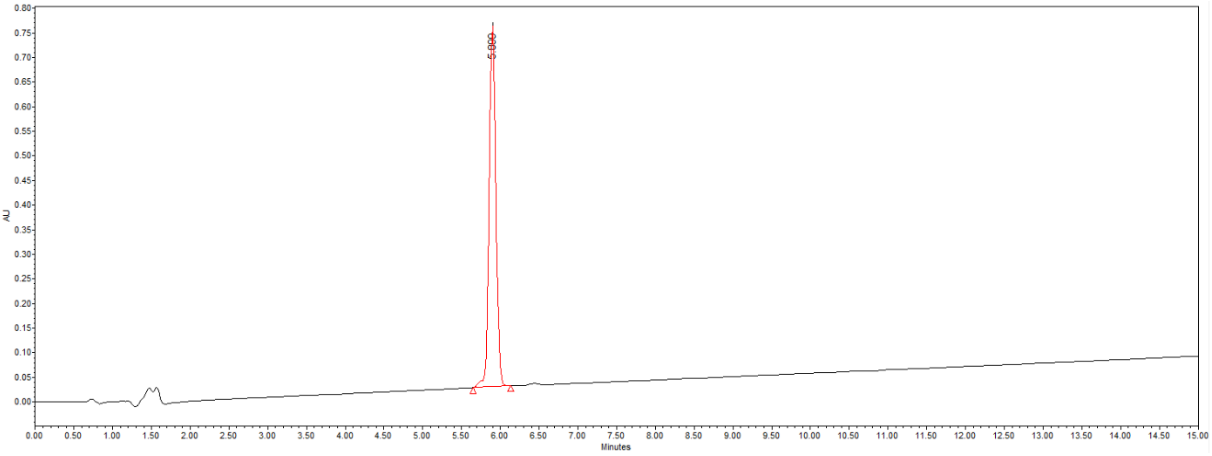


Figure S 24. RP-HPLC Spectrum of compound **S2**

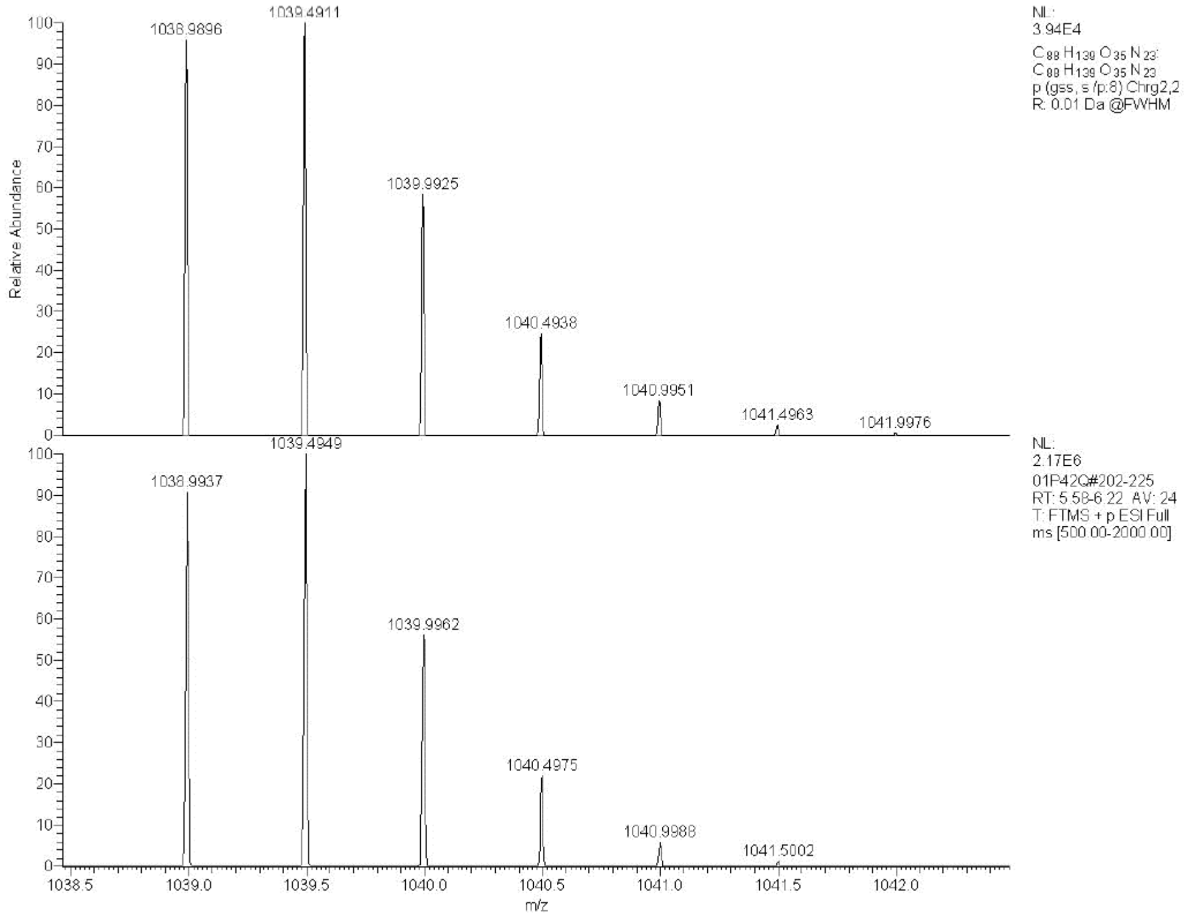


Figure S 25. HRMS spectrum of compound **S2**

## Compound **S3**

Prepared according general procedure **A** from **S2** (11.0 mg, 5.3 µmol) and **1** (1.4 mg, 1.20 µmol). The crude mixture was purified to afford the title compound as a white fluffy solid after lyophilization (7.7 mg, 0.82 µmol, 68%). HRMS (ESI$^+$-TOF) *m/z*: calcd for $C_{399}H_{630}N_{177}O_{178}$ [M+5H]$^{5+}$: 1887.5035, found 1887.5156; RP-HPLC: R$_t$ = 8.37 min (C18, λ = 214 nm, 0-30% B in 15 min).

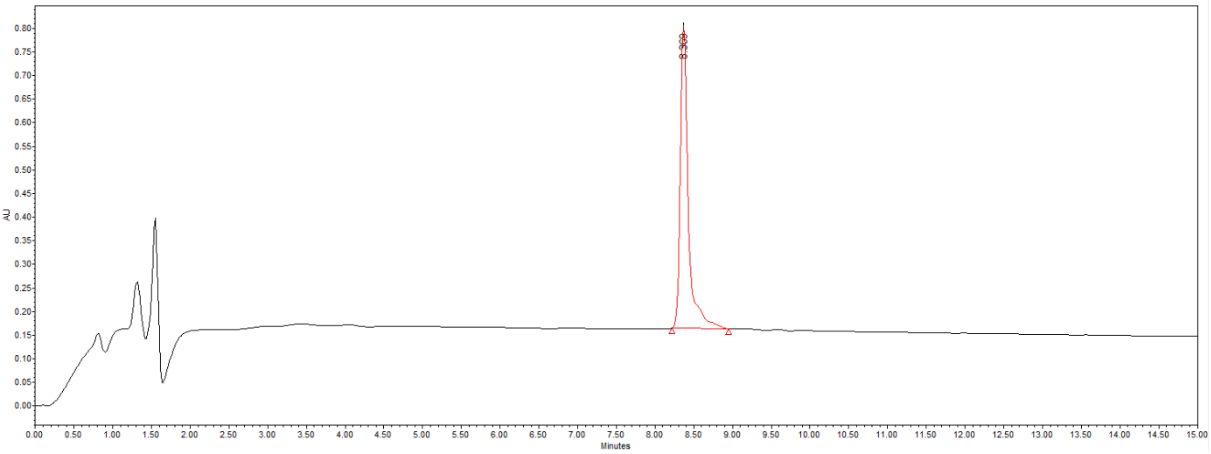


Figure S 26. RP-HPLC Spectrum of compound **S3**

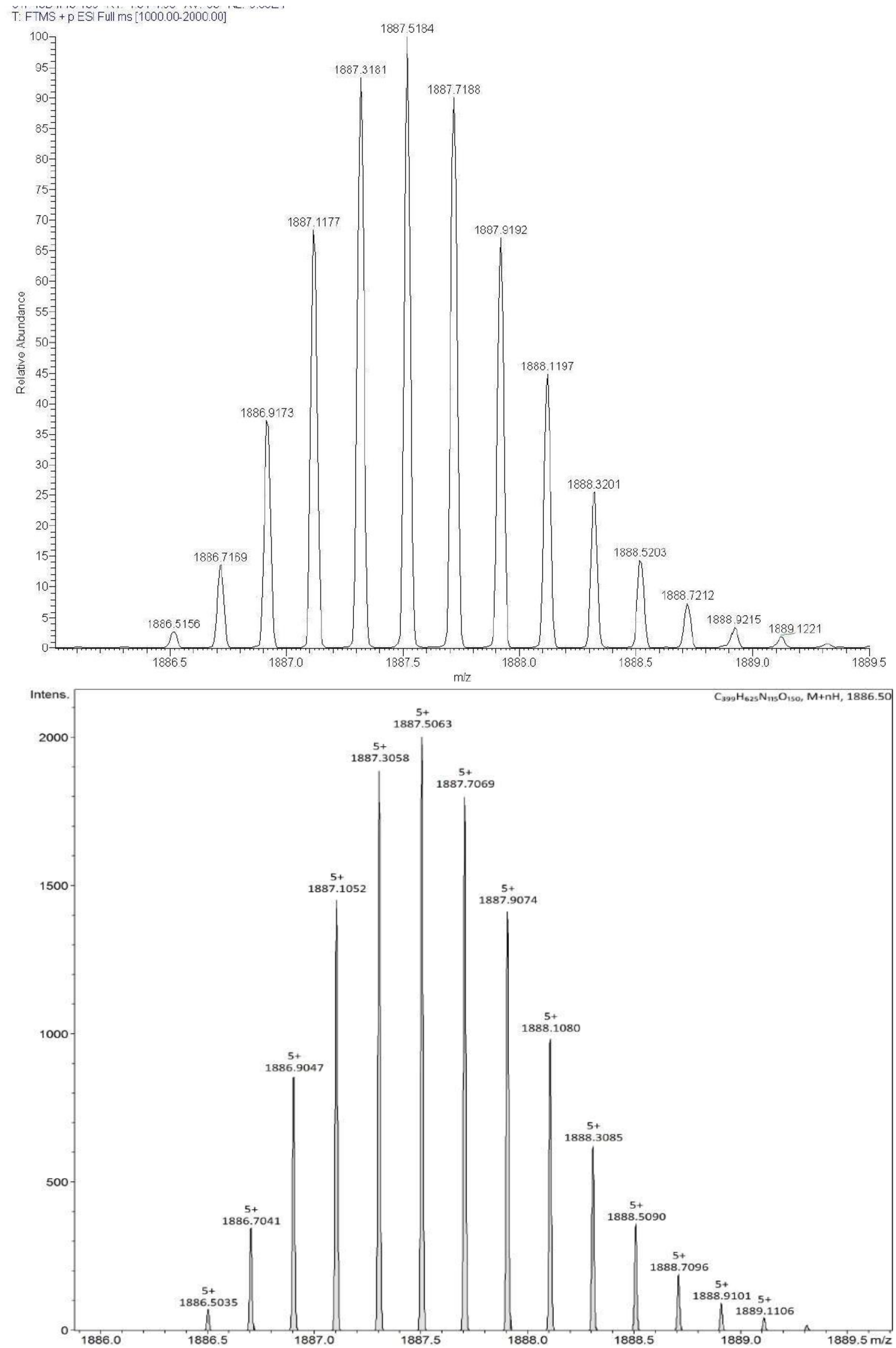


Figure S 27. HRMS spectrum of compound **S3**

Compound **S4**

Prepared according general procedure **B** from **S3** (7.0 mg, 0.74 µmol) and azidoacetic acid succinimide ester (0.2 mg, 1.1 µmol). The crude mixture was purified to afford the title compound as a white fluffy solid after lyophilization (6.6 mg, 0.69 µmol, 93%). HRMS (ESI$^+$-TOF) *m/z*: calcd for $C_{401}H_{631}N_{118}O_{151}$ [M+5H]$^{5+}$: 1903.1059, found 1903.1151; RP-HPLC: $R_t$ = 7.87 min (C18, λ = 214 nm, 5-40% B in 15 min).

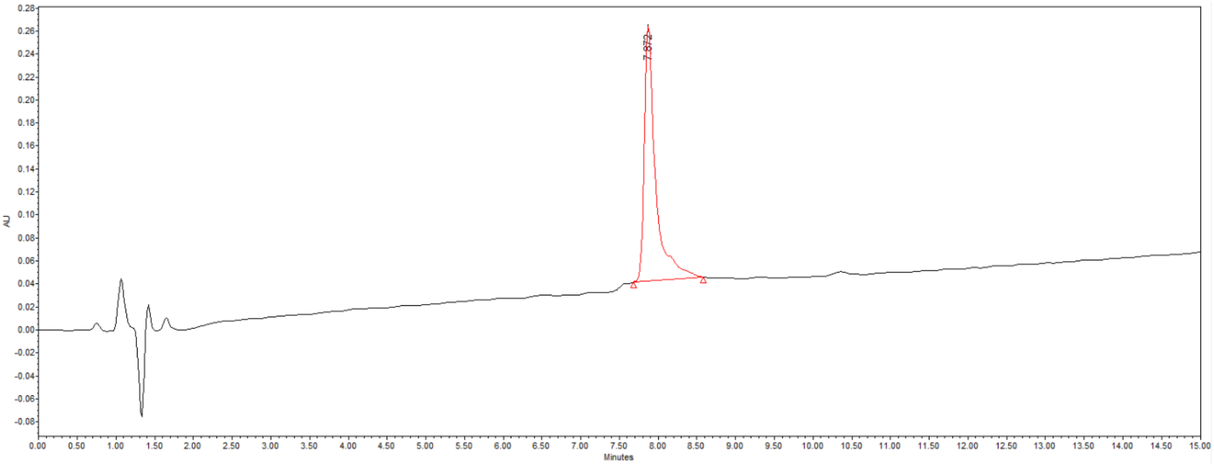


Figure S 28. RP-HPLC Spectrum of compound **S4**

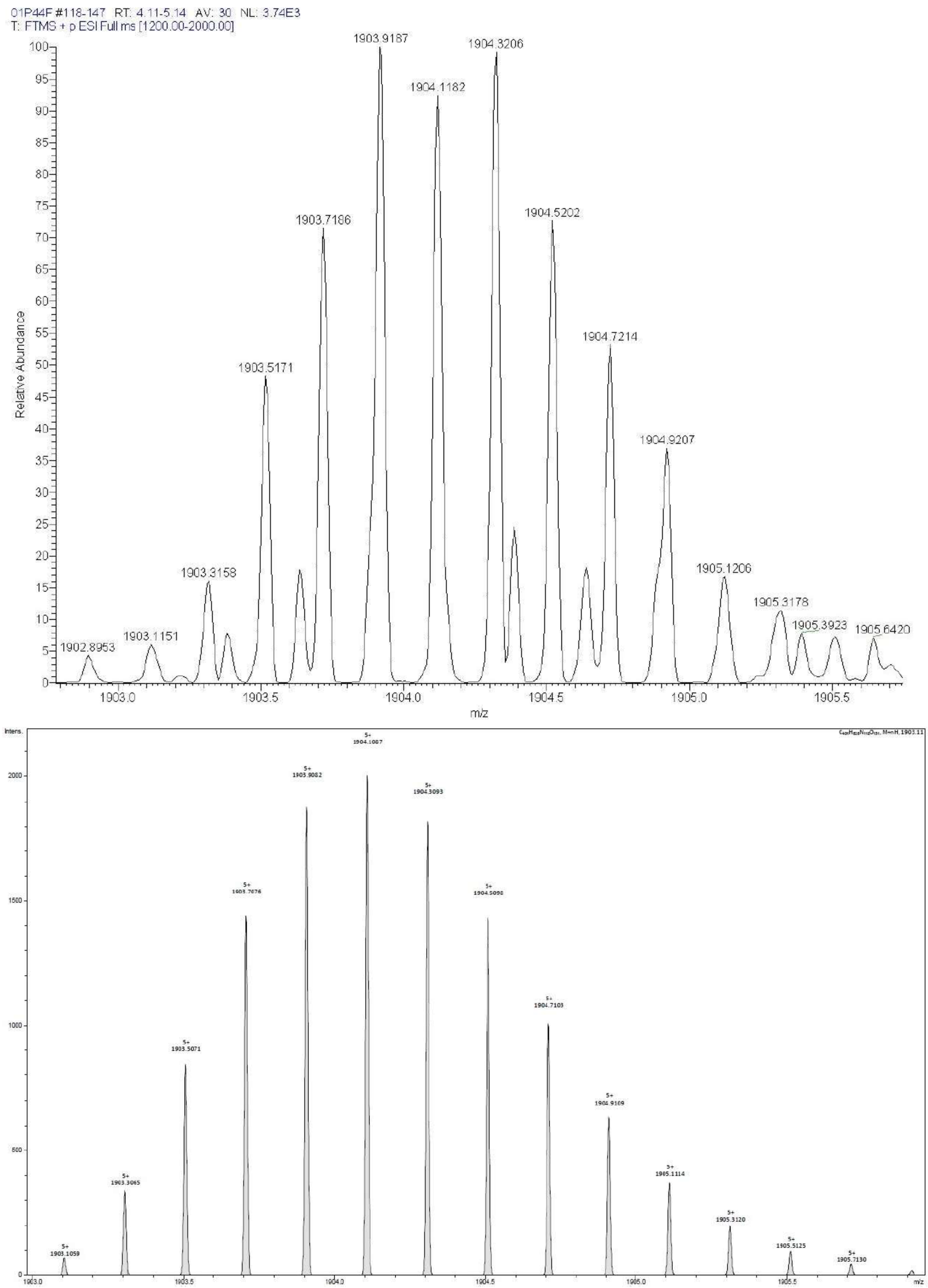


Figure S 29. HRMS spectrum of compound **S4**

## Compound **S5**

Prepared according general procedure **C** from **S4** (6.2 mg, 0.65 µmol) and **13** (2.6 mg, 0.65 µmol). The crude mixture was purified to afford the title compound as a white fluffy solid after lyophilization (6.9 mg, 0.51 µmol, 78%). HRMS (ESI$^+$-TOF) *m/z*: calcd for $C_{581}H_{887}N_{177}O_{194}$ $[M+7H]^{7+}$: 1922.0785, found 1922.0818; RP-HPLC: $R_t$ = 7.16 min (C18, λ = 214 nm, 5-40% B in 15 min).

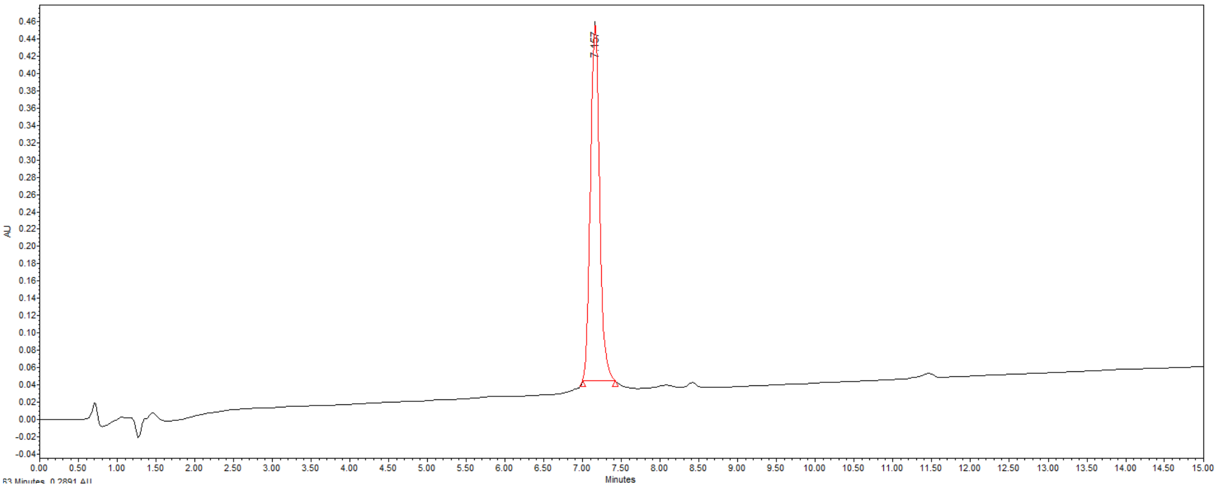


Figure S 30. RP-HPLC Spectrum of compound **S5**

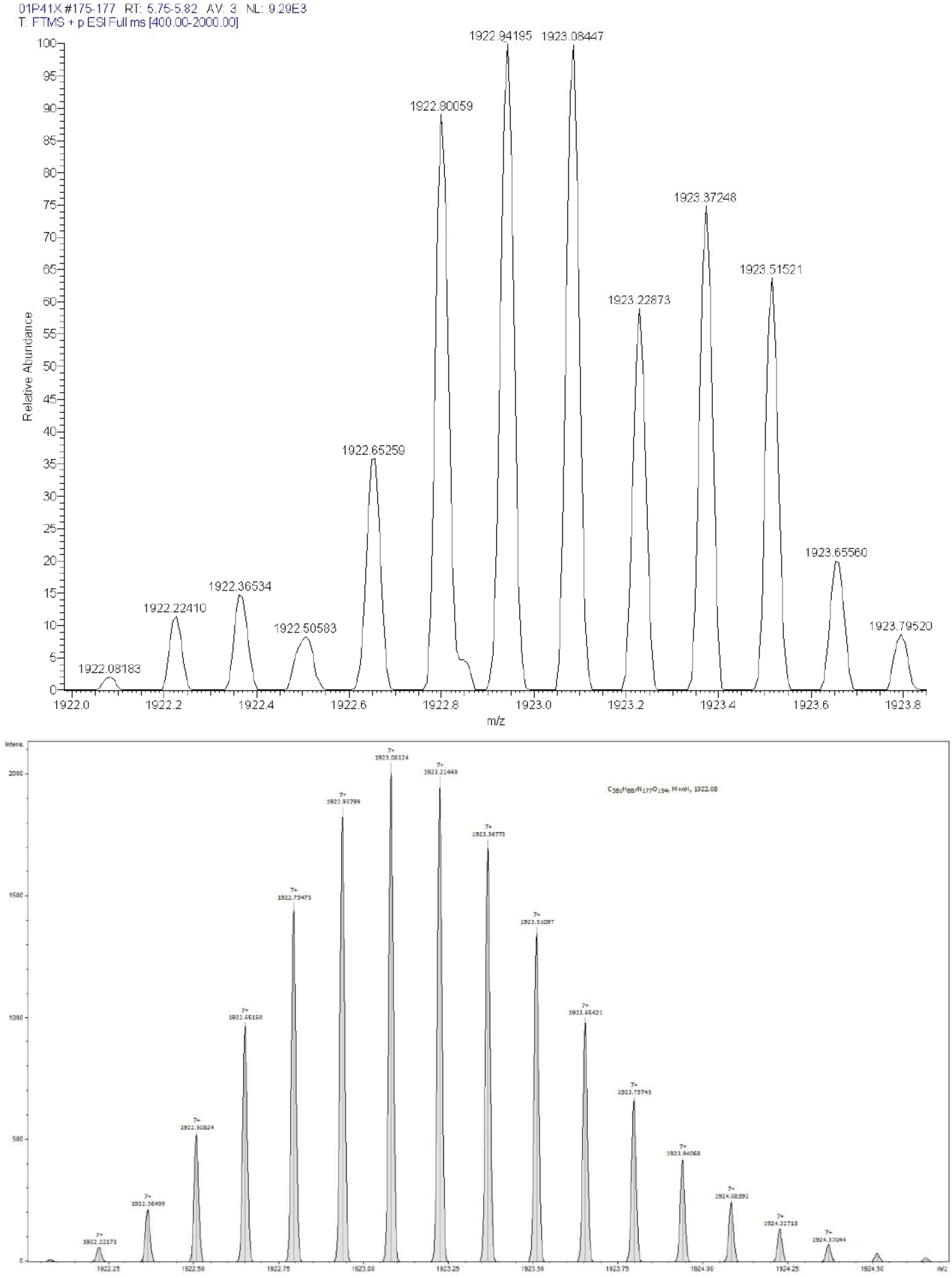


Figure S 31. HRMS spectrum of compound **S5**

# NMR spectra of final compounds

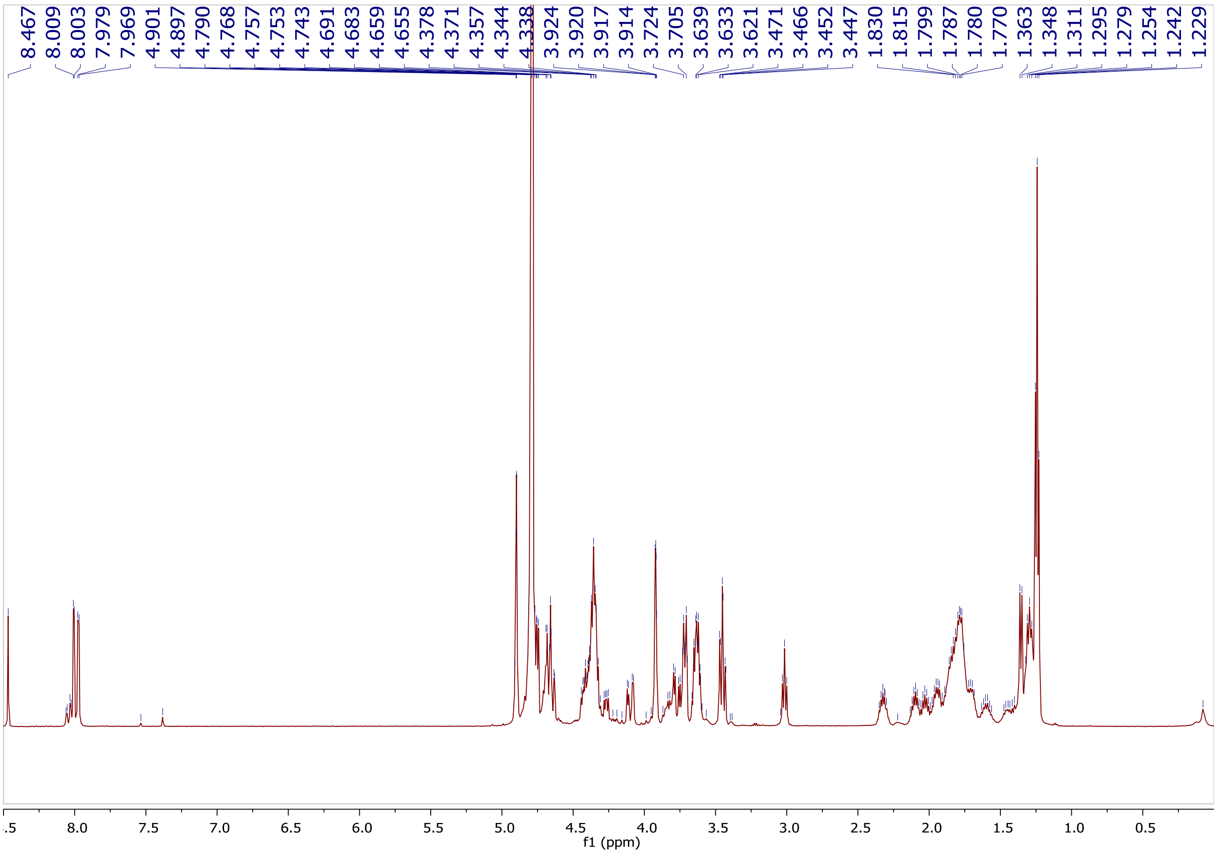


Figure S 32. $^{1}$H NMR ($D_2O$, 500 MHz) spectrum of compound **2**

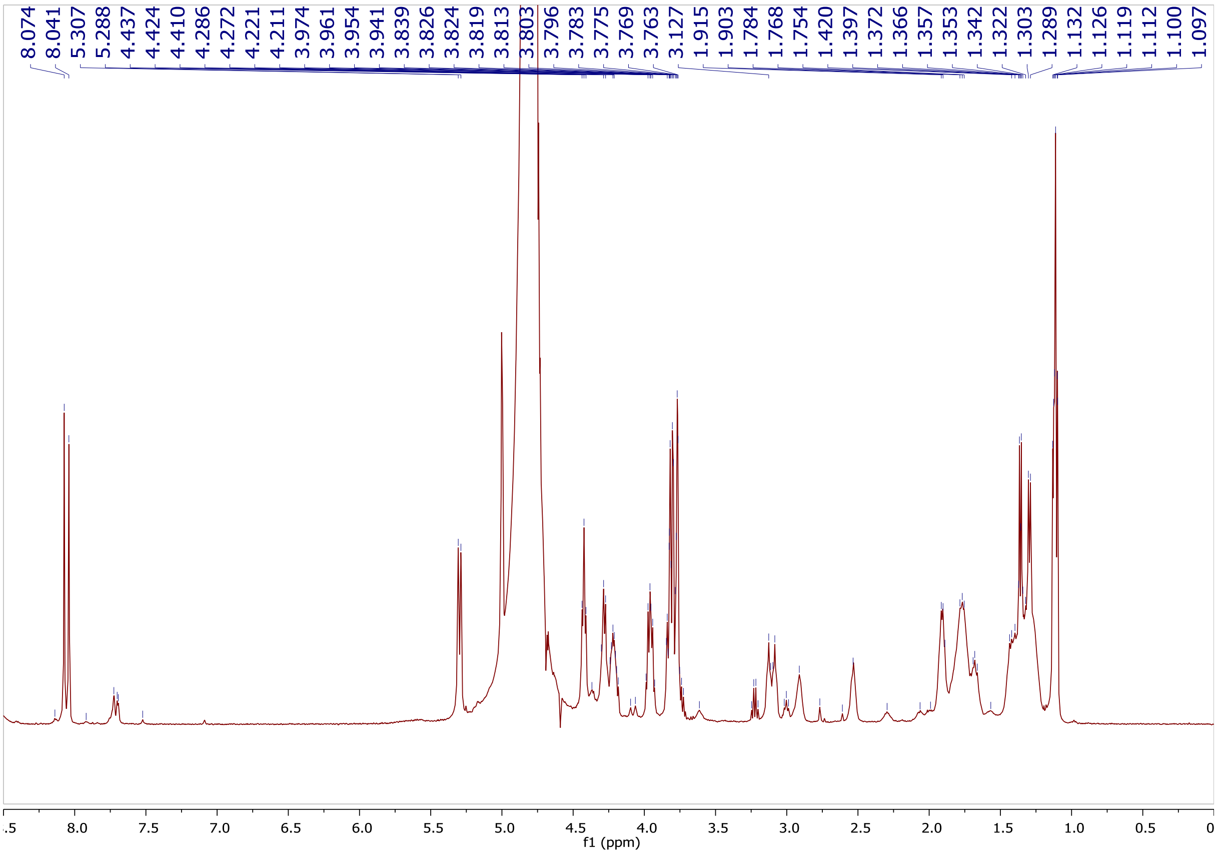


Figure S 33. $^{1}$H NMR ($D_2O$, 500 MHz) spectrum of compound **5**

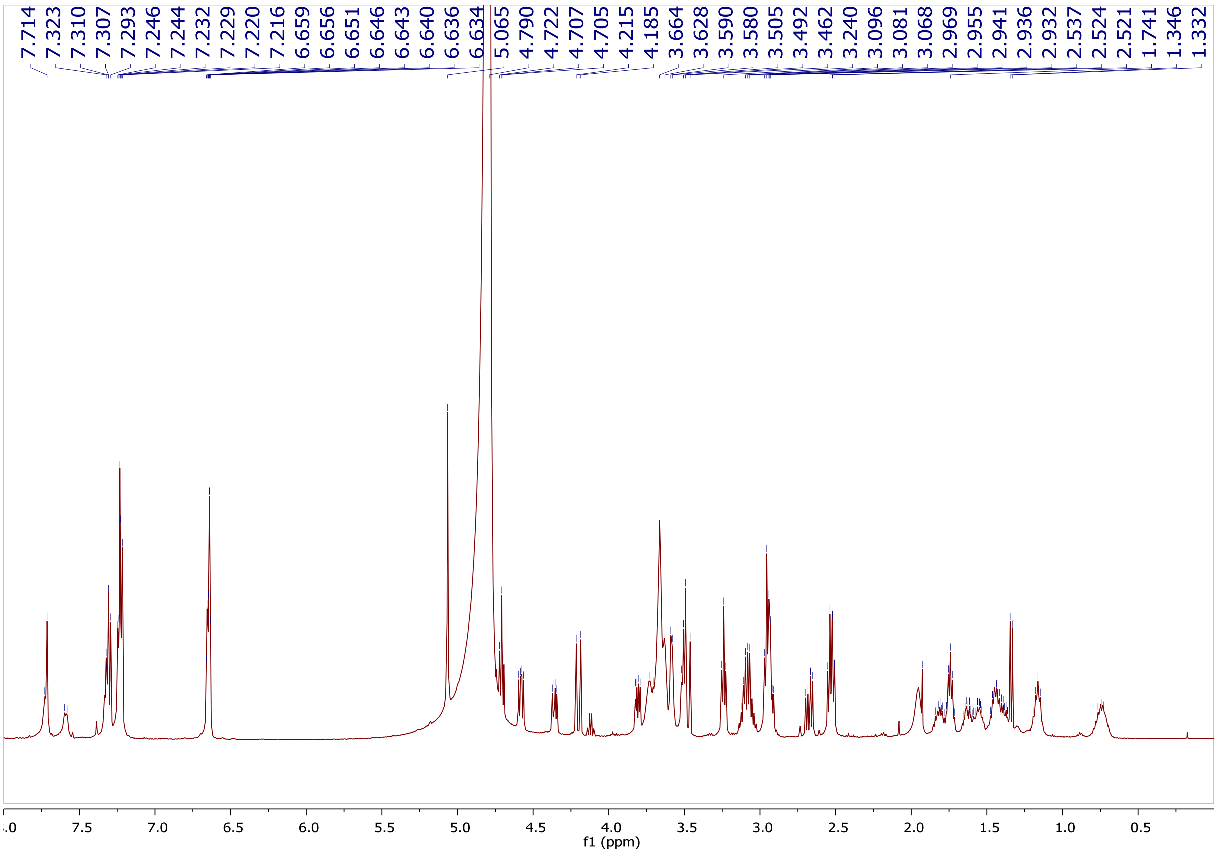


Figure S 34. $^{1}$H NMR ($D_2O$, 500 MHz) spectrum of compound **9**

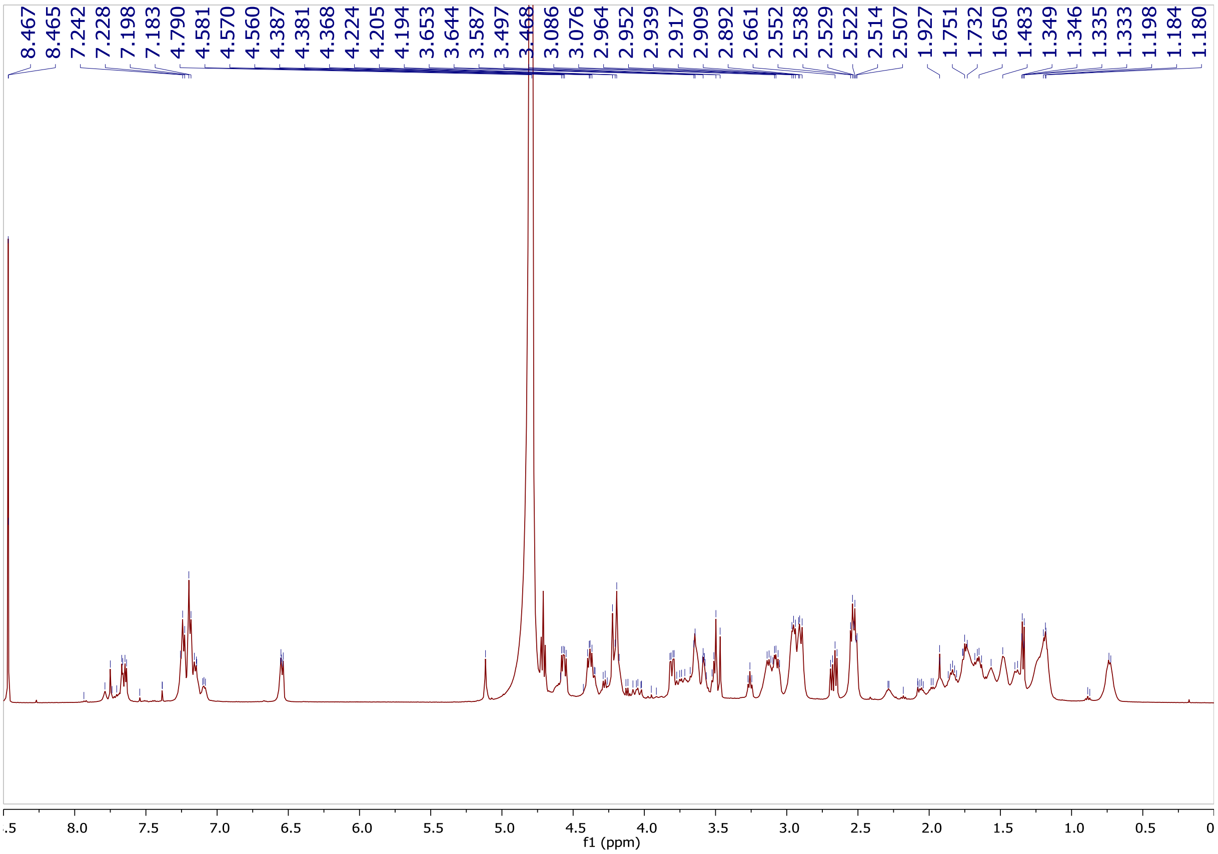


Figure S 35. $^{1}$H NMR ($D_2O$, 500 MHz) spectrum of compound **11**

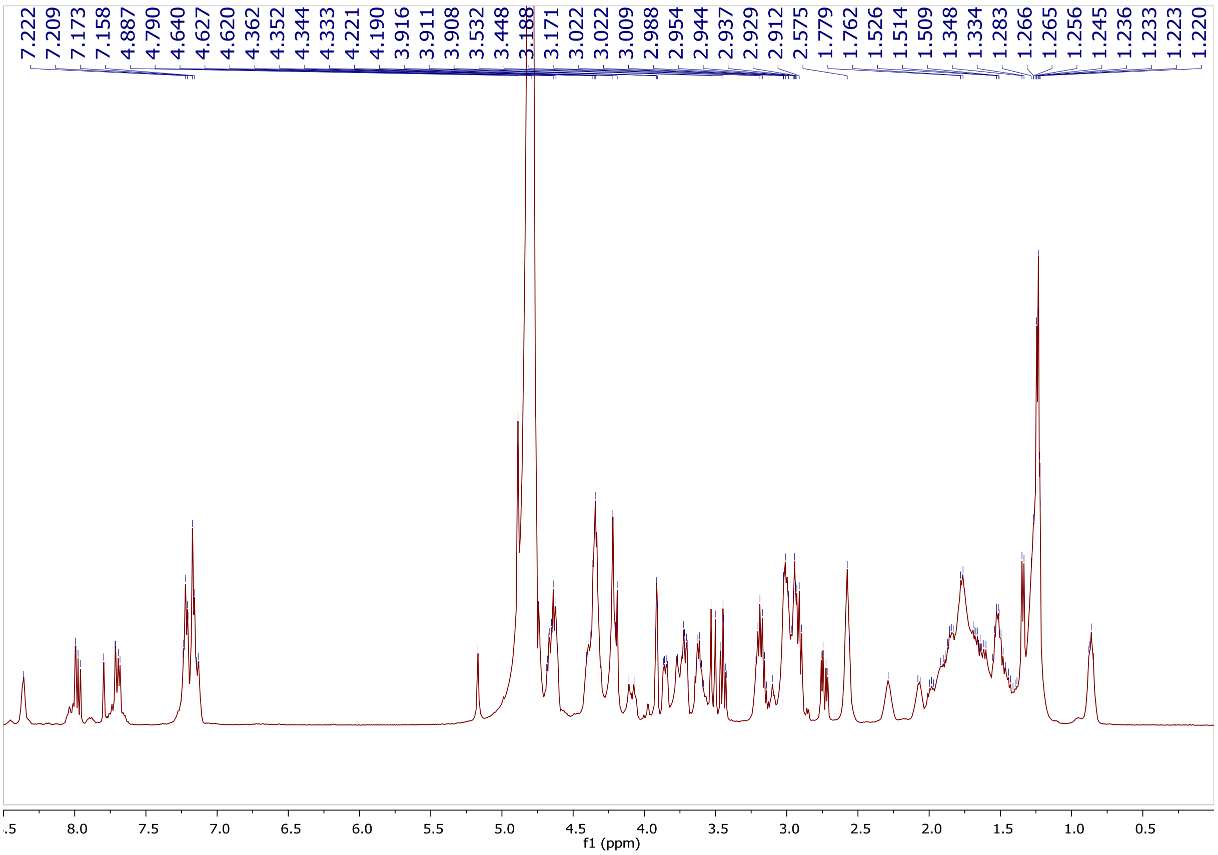


Figure S 36. $^{1}$H NMR ($D_2O$, 500 MHz) spectrum of compound **14**

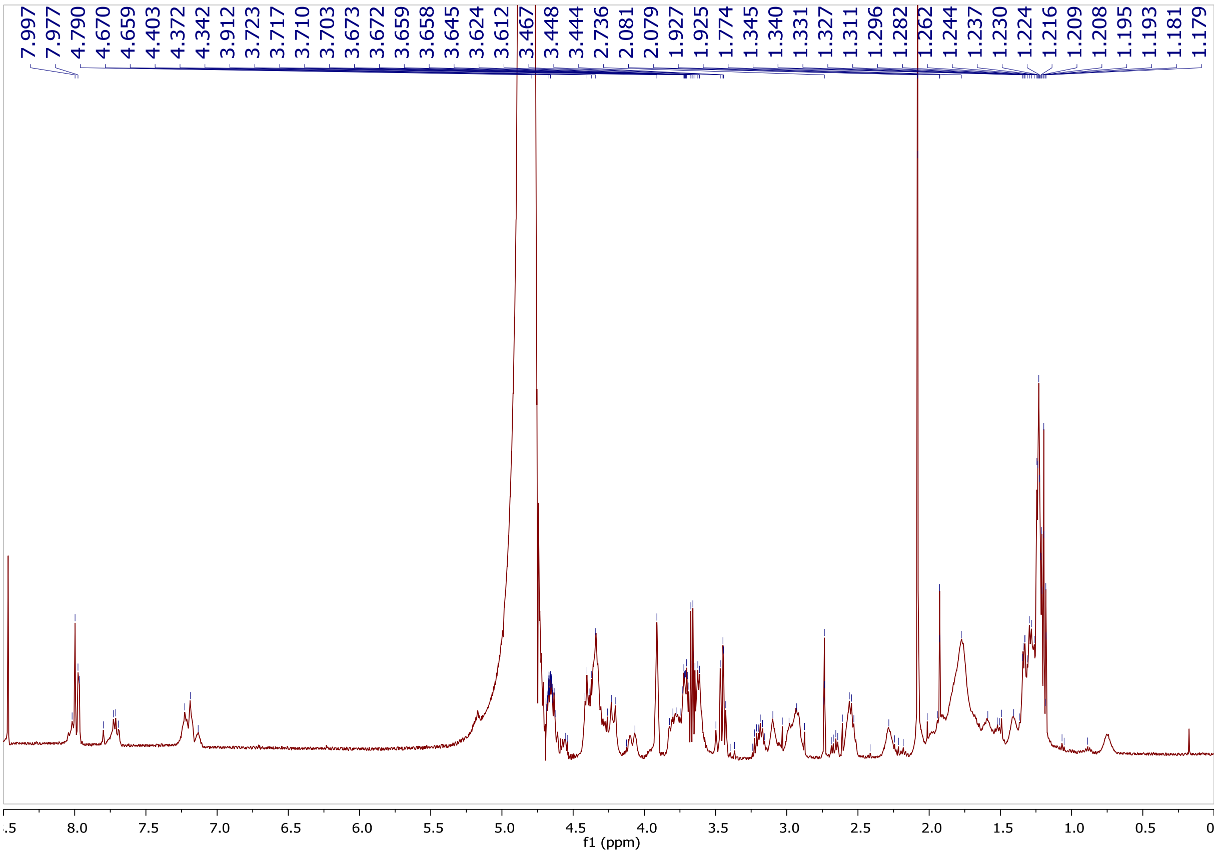


Figure S 37. $^{1}$H NMR ($D_2O$, 500 MHz) spectrum of compound **15**

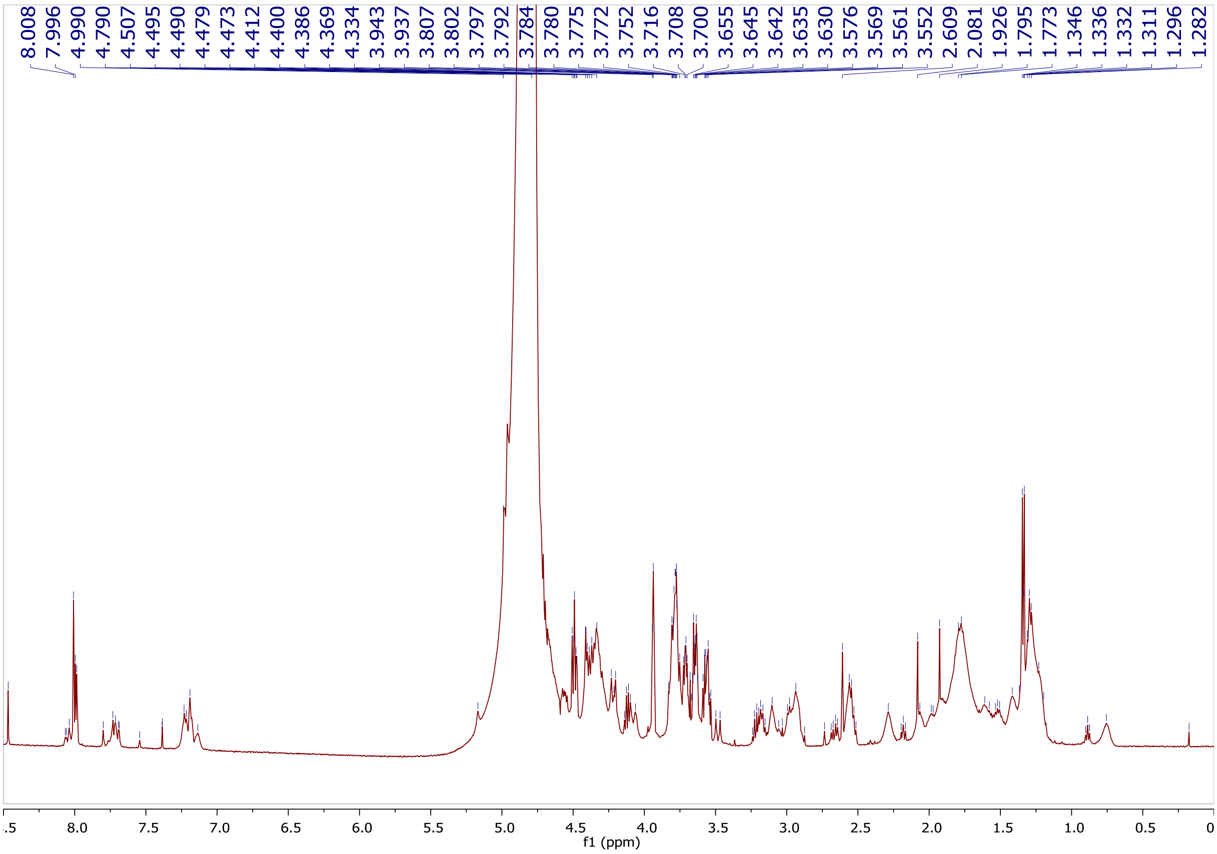


Figure S 38. $^{1}$H NMR ($D_2O$, 500 MHz) spectrum of compound **S5**

## ELISA assay

Two serial dilutions of the human serum were performed and added to PAA-Rha coated plate. Binding of the human anti-Rha antibodies from HS to the immobilized rhamnose was revealed by secondary antibodies Alexa Fluor$^{Tm}$ 488 goat anti-human IgG or IgM. Curves represent the fluorescence intensity versus the logarithm of the human serum percentage.

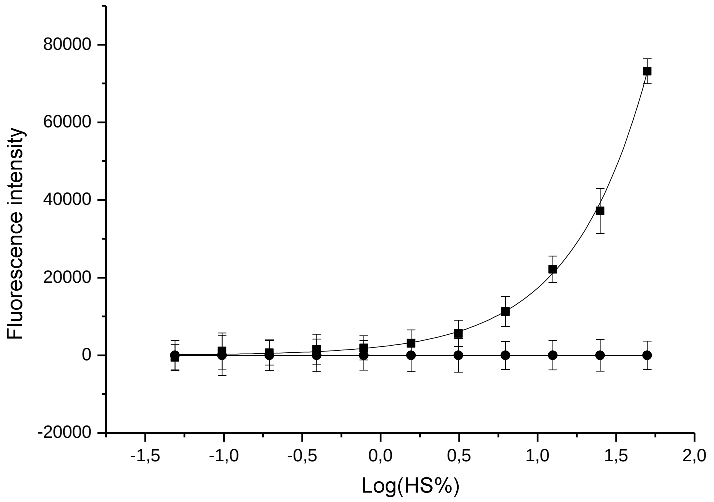


Figure S 39. Comparison of the levels of IgG and IgM anti-rhamnose antibodies in the human serum (HS).

## Confocal microscopy analysis of M21 cells incubated with 9 and 11

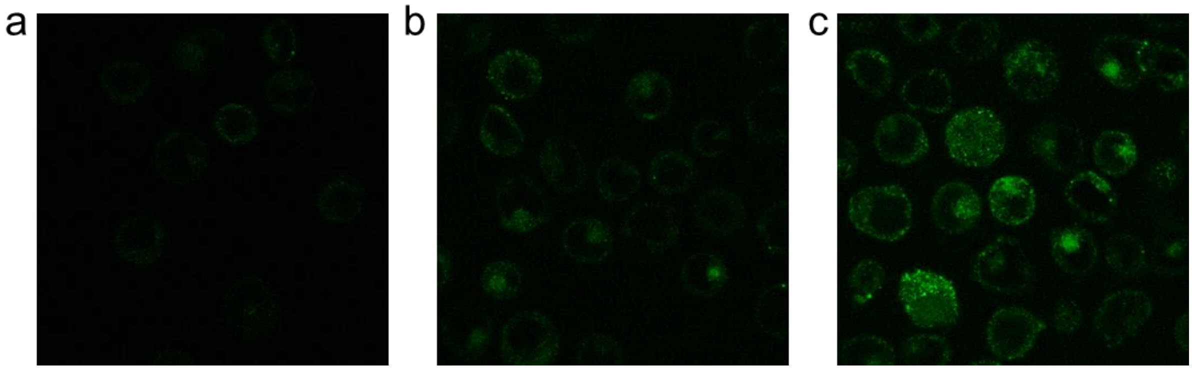


Figure S 40. Confocal fluorescence microscopy experiment after incubation of M21 with the TBM compounds **9** (b) and **11** (c) coupled with FITC. The M21 autofluorescence is observed in a.

## Cells αvβ3 integrins quantification

Near confluent cells were harvested, washed, counted and resuspended at a density of 1 x $10^6$ cells.mL$^{-1}$ in HBSS buffer. After a centrifugation step at 300g for 5 min, cells were resuspended in 200 µL of DMEM and 20 µL of a PE-coupled anti-CD51/CD61 antibody (5 µM) is added for 1h at 37°C. Cells were then washed with HBSS and

immediately analyzed using a BD LSR Fortessa flow cytometer (Becton Dickinson$^{TM}$). Data were analyzed using FCS express 6 software (De Novo Software$^{TM}$).

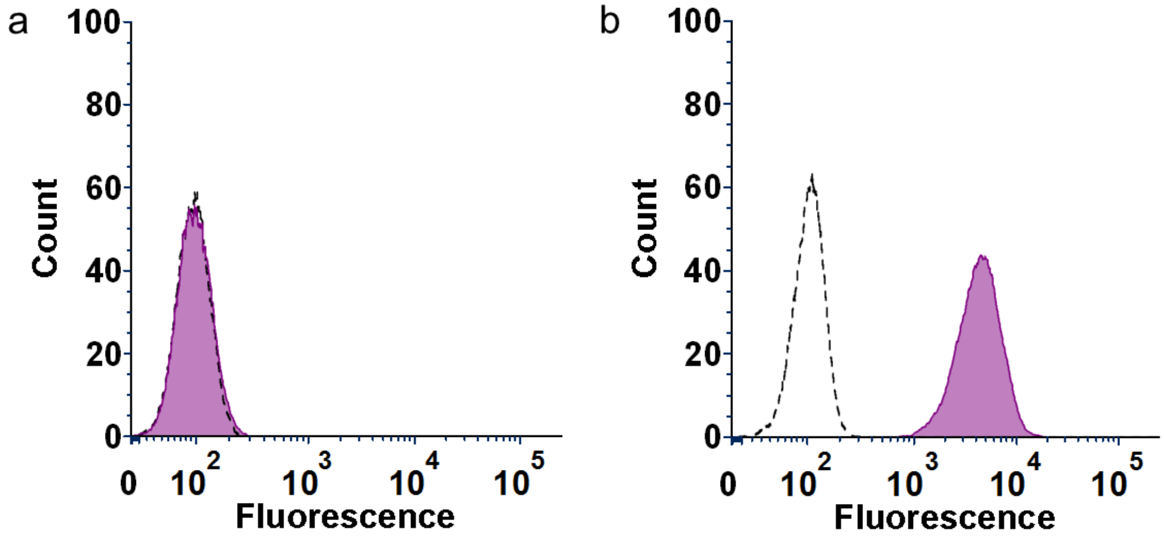


Figure S 41. Determination of the $\alpha_v\beta_3$ integrins concentration at the surface of a) M21-L and b) M21 cell lines by incubation of a PE-coupled anti-CD51/CD61 antibody (purple) . The fluorescence intensity is red with a flow cytometer. The cells autofluorescence are represented with dashed lines.

## Binding of human serum against galactosylated control

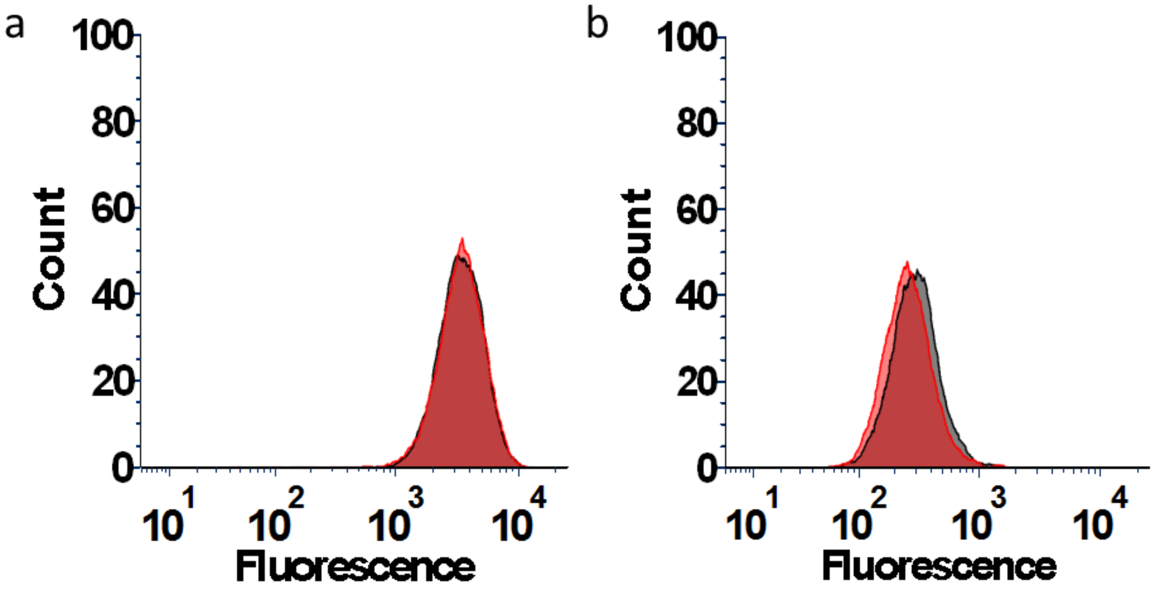


Figure S 42. Evaluation of the recruitment of antibodies present in human serum by flow cytometry with M21-L (a) and M21 (b) cell lines with ARM displaying Gal instead of Rha. Binding was revealed with Alexa FluorTM 488-coupled anti-human IgM antibody (1:400). Controls without the ARM molecule. Controls without the ARM molecule are represented in black

## References

(1) M. Galibert, L. Sancey, O. Renaudet, J. L. Coll, P. Dumy and D. Boturyn, *Org. Biomol. Chem.*, 2010, **8**, 5133-5138.
